# Transcriptome and Metabolome Analyses Reveals the Pathway and Metabolites of Grain Quality Under *Phytochrome B* in Rice (*Oryza sativa* L.)

**DOI:** 10.1186/s12284-022-00600-5

**Published:** 2022-10-27

**Authors:** Fei Li, Ye Liu, Xiaohua Zhang, Lingzhi Liu, Yun Yan, Xin Ji, Fanshu Kong, Yafan Zhao, Junzhou Li, Ting Peng, Hongzheng Sun, Yanxiu Du, Quanzhi Zhao

**Affiliations:** Henan Key Laboratory of Rice Biology, Collaborative Innovation Center of Henan Grain Crops, Henan Agricultural University, No. 15, Longzihu University Park, Zhengdong New Area, Zhengzhou, China

**Keywords:** *OsPHYB*, Chalkiness, Grain size, Transcriptome, Metabolome, Correlation analysis

## Abstract

**Background:**

Grain size and chalkiness is a critical agronomic trait affecting rice yield and quality. The application of transcriptomics to rice has widened the understanding of complex molecular responsive mechanisms, differential gene expression, and regulatory pathways under varying conditions. Similarly, metabolomics has also contributed drastically for rice trait improvements. As master regulators of plant growth and development, phys influence seed germination, vegetative growth, photoperiodic flowering, shade avoidance responses. *OsPHYB* can regulate a variety of plant growth and development processes, but little is known about the roles of rice gene *OsPHYB* in modulating grain development.

**Results:**

In this study, rice *phytochrome B* (*OsPHYB*) was edited using CRISPR/Cas9 technology. We found that *OsPHYB* knockout increased rice grain size and chalkiness, and increased the contents of amylose, free fatty acids and soluble sugar, while the gel consistency and contents of proteins were reduced in mutant grains. Furthermore, *OsPHYB* is involved in the regulation of grain size and chalk formation by controlling cell division and complex starch grain morphology. Transcriptomic analysis revealed that loss of *OsPHYB* function affects multiple metabolic pathways, especially enhancement of glycolysis, fatty acid, oxidative phosphorylation, and antioxidant pathways, as well as differential expression of starch and phytohormone pathways. An analysis of grain metabolites showed an increase in the free fatty acids and lysophosphatidylcholine, whereas the amounts of sugars, alcohols, amino acids and derivatives, organic acids, phenolic acids, alkaloids, nucleotides and derivatives, and flavonoids decreased, which were significantly associated with grain size and chalk formation.

**Conclusions:**

Our study reveals that, *OsPHYB* plays an important regulatory role in the growth and development of rice grains, especially grain size and chalkiness. Furthermore, *OsPHYB* regulates grain size and chalkiness formation by affecting gene metabolism interaction network. Thus, this study not only revealed that *OsPHYB* plays a vital role in regulating grain size and chalkiness of rice but reveal new functions and highlighted the importance and value of *OsPHYB* in rice grain development and provide a new strategy for yield and quality improvement in rice breeding.

**Supplementary Information:**

The online version contains supplementary material available at 10.1186/s12284-022-00600-5.

## Background

Rice (*Oryza sativa* L.) is consumed by more than half the world’s population, and the improvement in varieties and cultivation techniques has significantly improved the yield and quality of rice (Tang et al. 2019). Moreover, rice is an important source of protein, energy, fibers, minerals, and bioactive compounds in many countries around the world (Ferreira et al. 2021). As living standards improve, consumer expectations of rice quality also increase. Grain chalkiness is a critical trait that determines the quality of rice (Hoshikawa 1989). Grain chalkiness in rice refers to the opaque part of endosperm, and its formation is controlled by multiple factors, including starch synthesis, the structure and arrangement of starch granules, phytohormones, and various external stresses during grain filling (Nevame et al. 2018; Yang et al. 2022). Mutations have been introduced to many of the genes involved in starch synthesis and carbon metabolism, such as glycolysis. Such genes include *SSI*, *Wx*, *SSIIIa*/*FLO5*, *BEIIb*, *OsAPL2*, *OsPK3* and *OsPPDKB* that result in a chalky phenotype (Hu et al. 2020; Xie et al. 2021; Zhang et al. 2022). Moreover, other factors related to the development of amyloplasts, such as *FLO2*, *FLO7*, *SSG4* and *ISA1*, also play important roles in the formation of chalkiness (Matsushima et al. 2014; Xie et al. 2021). Protein and lipid metabolism also affect the formation of chalkiness (Lin et al. 2010; Wang et al. 2015a; Wu et al. 2019; Yu et al. 2021a).

In addition, multiple complex regulatory pathways are involved in grain size and the formation of chalkiness. Higher levels of auxin, cytokinins (CKs) and gibberellins (GAs) could result in more chalkiness, while brassinosteroids (BRs) could reduce chalkiness (Zhang et al. 2020). Changes in the contents of abscisic acid (ABA) and ABA/GA had significant effects on grain filling and chalkiness (Xiao et al. 2017). The formation of grain chalkiness can also be influenced by various external stresses during the grain-filling stage. Recent studies showed that reactive oxygen species (ROS) may play a critical role in the regulation of rice endosperm chalkiness (Lin et al. 2022; Liu et al. 2010; Xu et al. 2010). Grain size is directly related to changes in the composition, growth, and accumulation of the endosperm because it affects yield and quality, which are usually negatively correlated with each other (Wang et al. 2012). Genes such as *GIF1*, *qGW8*, *GL7*, *OsMADS1*, can regulate the quality of rice while regulating grain size (Liu et al. 2018; Wang et al. 2008, 2012, 2015b). Although some progress had been made in the research on chalk formation, particularly grain size, the research on rice grain size and chalk formation still merits further study, particularly the role of phytochromes.

The phytochrome family primarily senses red and far-red light to regulate a range of developmental processes throughout the life cycle of plants. The phytochrome gene family in rice is composed of three members that include *PHYA*, *PHYB* and *PHYC* (Sun et al. 2017). Current studies have shown that rice PHYB can adjust the de-yellowing, leaf angle, flowering stage, hypocotyl elongation, shade avoidance, and fertility of seedlings by receiving red light (Osugi et al. 2011; Takano et al. 2009, 2005). In addition, PHYA has unique and overlapping roles with PHYB in the regulation of photosynthesis in rice. The light-regulated overexpression of an Arabidopsis *PHYA* gene in rice has been reported to alter plant architecture and increase grain yield (Garg et al. 2006). By regulating the photosynthetic process, the phytochrome system also influences the source-sink relationship, grain quality, and yield. However, the regulation of PHYB on rice grain characters and quality has not been reported.

In this study, rice *phytochrome B* (*OsPHYB*) was edited using CRISPR/Cas9 technology. After screening and identification, homozygous *OsPHYB* loss-of-function mutants were obtained, and their grain shape and quality traits were determined to study the effect of *OsPHYB* on grain traits, and transcriptomic and metabolomic analyses were utilized to investigate the molecular mechanism underlying the variation in traits.

## Results

### OsPHYB Mutation Increased the Grain Size and Formation of Chalkiness in Rice

**Fig. 1:**
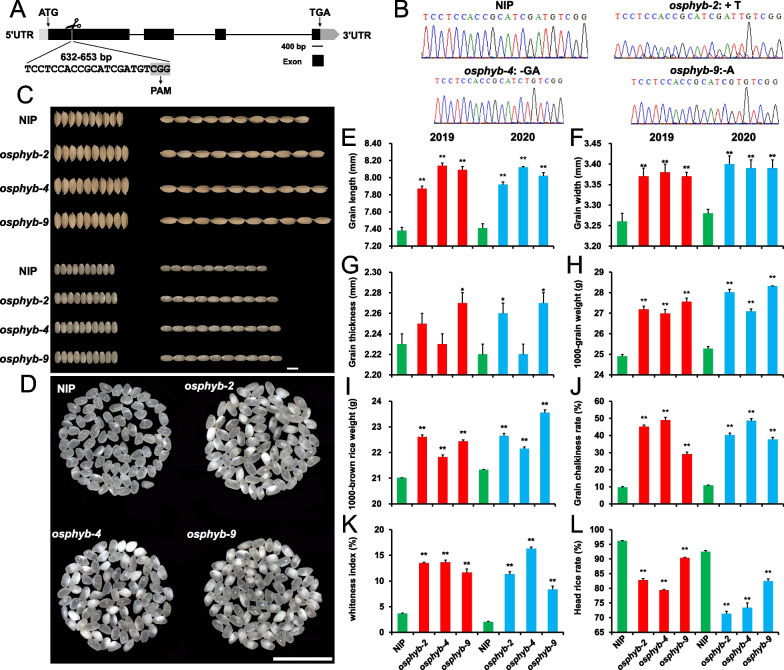
Loss of *OsPHYB* function resultes in larger grain size and increases chalkiness in rice. **A** Location of *OsPHYB* knockout target sequence. **B** The sequencing results of target sequence for three T_3_ generation pure and knockout lines of *OsPHY*B. **C**–**D** Grain size comparison of *osphyb* mutant and NIP plants in rice and brown rice. Bars = 5 mm. **E**–**I** Statistics of grain length, grain width, grain thickness 1000-grain weigh and brown rice 1000-grain weight of *osphyb* mutant and NIP rice in 2019 and 2020. *P* < 0.05, ** *P* < 0.01. **J**–**L** Statistics of *osphyb* mutant and NIP grain chalkiness rate, whiteness index and head rice rate in 2019 and 2020. *P < 0.05, ** *P* < 0.01

To investigate the effects of *OsPHYB* on grain size, yield, and quality of rice, we used CRISPR/cas9 to edit the first exon of *OsPHYB* to obtain *OsPHYB* mutants (Figs. 1A and Additional file 1: S1A). Three independent lines of homozygous knockout plants (T_3_ generation), *osphyb-2* (single base “T” insertion), *osphyb-4* (double base “GA” deletion) and *osphyb-9* (single base “A” deletion) were adopted in the study (Fig. 1B). All three types of base mutations resulted in the termination of translation of their amino acids after the putative PAM domain, resulting in the loss of function of OsPHYB (Additional file 1: Fig. S1B, C).

Two-year field data analysis showed that the deletion of *OsPHYB* significantly improved the grain size and 1,000-grain weight of rice, particularly the grain length and grain width. Compared with NIP, the average grain length, grain width, 1000-grain weight and 1000-brown rice weight increased by 8.54%, 3.47%, 9.74% and 6.43%, respectively (Fig. 1C–I). In addition, the *OsPHYB* mutants also increased the grain chalkiness of transgenic plants, with an average increase of 305.65% and 371.61% in chalky grain rate and chalkiness, respectively, compared with those of NIP (Fig. 1D, J–K). The head rice rate of *osphyb* was reduced by an average of 15.24% compared with that of NIP (Fig. 1L). In addition, compared with NIP, the *osphyb* mutant also significantly decreased plant height, tiller number, seed setting rate, panicle length, number of primary branches and secondary branches (Additional file 1: Fig. S2A–F, H–K). It was also found that the flowering phase of the *osphyb* mutant was about 3 weeks earlier relative to NIP (Additional file 1: Fig. S2A, G). The contents of nutrients in the NIP and *OsPHYB* deletion mutant rice were also determined. The knockout of *OsPHYB* significantly increased the contents of amylose, free fatty acids (FFA), and soluble sugar (*P* < 0.01), whereas the content of protein and gel consistency significantly decreased (*P* < 0.01). The contents of amylopectin and soluble starch increased slightly, but the results were not significant (Table 1).

**Table 1:** Analysis of seed quality traits parameters of *osphyb* and NIP

Lines	Amylose contents (mg/g)	Amylopectin contents (mg/g)	Starch contents (mg/g)	Soluble sugar contents (mg/g)	Protein contents (mg/g)	Free fatty acid contents (mg/g)	Gel consistency (mm)
NIP	16.13 ± 0.28	256.32 ± 35.09	469.63 ± 22.86	5.92 ± 0.04	302.39 ± 2.49	10.00 ± 0.46**	86.25 ± 0.72
*osphyb-2*	21.39 ± 0.09**	260.44 ± 31.64	511.29 ± 33.04	6.87 ± 0.02**	204.75 ± 9.66**	13.20 ± 0.46**	76.81 ± 0.99**
*osphyb-4*	20.03 ± 0.72**	271.15 ± 17.18	524.54 ± 26.17	6.44 ± 0.10**	266.54 ± 5.22**	13.33 ± 0.48**	71.54 ± 0.95**
*osphyb-9*	21.26 ± 0.40**	270.88 ± 27.48	499.49 ± 10.10	6.22 ± 0.03**	207.30 ± 3.66**	12.80 ± 0.23**	69.17 ± 1.38**

*P < 0.05, **P < 0.01

### OsPHYB Regulates the Number of Grain Cells and Complex Starch Granule Morphology in Rice

**Fig. 2:**
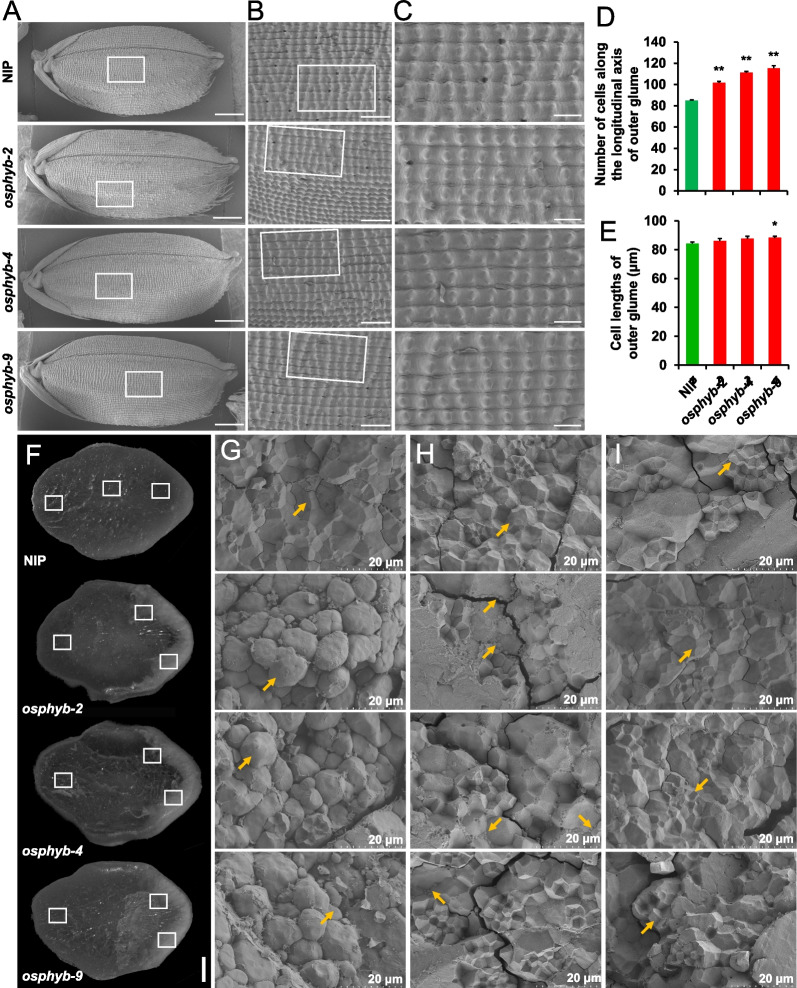
Scanning electron microscopy analysis of mature grains and grain cross-sections of *OsPHYB* mutants and NIP. **A** Scanning electron photographs of the outer glume of mature seeds in NIP and the *osphyb* mutant. Bars = 1 mm. **B** Scanning electron microscopy observation of lemma. Bars = 250 μm. **C** Magnified view of the outer surface area boxed in (**B**). Bars = 100 μm. **D**–**E** Cell number and cell length of fully mature seeds along the longitudinal axis. *P < 0.05, ** *P* < 0.01. **F** Comparison of grain cross sections in *osphyb* and NIP. Bar = 1 mm. **G**–**I** Scanning electron microscopy analysis of different parts of grain cross sections in *osphyb* and NIP. Bars = 20 μm

To elucidate how *OsPHYB* regulates grain length, we investigated cell size and cell number in the outer epidermis of spikelets using SEM (Fig. 2). It was observed that in the *osphyb* mutant, more cells were observed along the longitudinal axis of the grain lemma, a 28.76% increase relative to the number of NIP cells, while the size of the cells did not change significantly (Fig. 2A–E). Grains produced from the *osphyb* transgenic plants had a highly chalky appearance compared with those from NIP (Fig. 1D and 2F). Notably, a cross-sectional analysis showed that the belly region of *osphyb* mutant grain appeared floury-white, while the inner endosperm was translucent as in the NIP endosperm (Fig. 2F). Moreover, the abdominal endosperm cells of *osphyb* mutants were filled with loosely arranged complex starch granules with larger spaces, which were distinct from the densely arranged endosperm cells of NIP and exhibited large spherical shapes (Fig. 2G). Endosperm cells on the other side of *osphyb* were consistent with irregular polyhedral starch granules compared with those of NIP (Fig. 2J). At the excessive position of the farinaceous to hyaline regions, a very small number of small, rounded starch complex granules were observed in the *osphyb* (Fig. 2H).

### Overview of Transcriptional Profiles of All Rice Samples

**Fig. 3:**
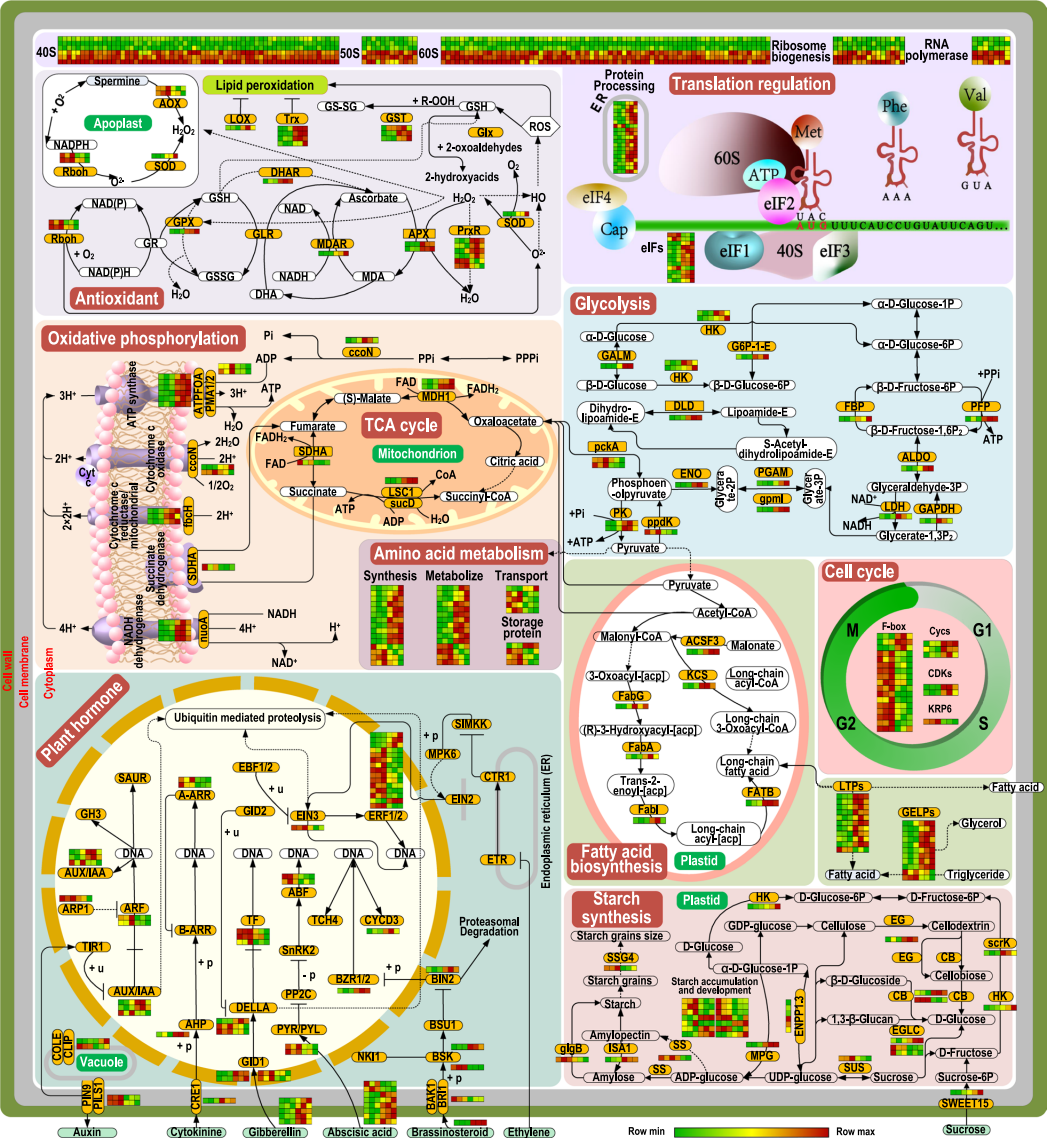
Schematic model of gene regulatory networks for the grain size and chalkiness formation of *osphyb*. Pathway: Glycolysis, TCA cycle, starch synthesis, amino acid metabolism, fatty acid synthesis, plant hormones, oxidative phosphorylation, antioxidation, translation regulation. Genes encoding enzyme (s) are indicated in orange boxes, metabolites and other materials in white boxes. The expression patterns of the genes encoding corresponding enzyme (s) are indicated above or below the arrows. Each row (6 boxes) represents a DEG. Six boxes in each row represent 3 biological repeats of one gene in NIP (first three boxes) and *osphyb* (last three boxes), respectively. The enriched DEGs boxes for 40S, 50S, 60S, Ribosome biogenesis and RNA polymerase are rotated 90° clockwise. Log_2_ (fold changes) are represented by a colour scale from green (down-regulated) to red (up-regulated)

Grain size and quality are complex quantitative traits that are regulated by multiple genes. To study the gene regulatory network of *OsPHYB* on grain size and quality in more detail, a transcriptomic analysis of NIP and *OsPHYB* mutant transgenic grains was performed. A fragments per kilobase of transcript per million mapped fragments (FPKM) box plot, FPKM density distribution, biological repeats (Pearson’s correlation coefficient), and differentially expressed gene (DEG) different heat map results are shown in Additional file 1: Fig. S3. The volcano plot and differential heatmap of the DEGs showed that 51.86% of the 2137 differential genes were up-regulated, whereas 48.14% were down-regulated (Additional file 1: Fig. S3D, E). The carbohydrate transport and metabolism, fatty acid synthesis and metabolism, oxidative phosphorylation, multiple amino acid synthesis, cell cycle, ribosome, RNA polymerase, protein translation and modification, and various organic acid metabolisms, among others, were annotated or enriched between *osphyb* and NIP material based on KOG annotation and KEGG pathway enrichment (Additional file 1: Fig. S4). We mainly interpret carbon metabolism-related pathways that primarily included glycolysis, the tricarboxylic acid (TCA) cycle, amino acid metabolism and starch synthesis along with fatty acid synthesis, oxidative phosphorylation, antioxidant pathways and cyclins, plant hormone signal and transduction, which involved in regulating rice grain quality and size mediated by *OsPHY*B (Fig. 3). The expression of transcriptome genes was further confirmed by qRT-PCR. The samples we used for qRT-PCR were the same transgenic lines as the transcriptome, which were rice grain (12 days after flowering) samples of the same period in the second year. qRT-PCR quantitative validation results are shown in Additional file 1: Fig. S5.

### OsPHYB Mutation Results in Differential Expression of Multiple Carbon Metabolism Pathway Genes

In this study, the pathway involved in glycolysis was found to be activated by up-regulating the levels of expression of genes encoding the enzymes involved in glycolysis, including hexokinase, phosphoglucomutase, phosphofructokinase, aldolase, lactate dehydrogenase, glyceraldehyde-3-phosphate dehydrogenase, enolase, and pyruvate kinase, and decreased the levels of expression of gluconeogenesis rate-limiting enzymes, such as phosphoenolpyruvate carboxykinase, and one pyruvate phosphate dikinase gene (PPDK) in *osphyb* compared with those in NIP (Fig. 3 and Additional file 2: Table S2). The TCA cycle is the final metabolic pathway of the three major nutrients (carbohydrates, lipids, and amino acids) and also the hub of the connections between carbohydrate, lipid, and amino acid metabolism. The succinyl-CoA ligase (*CoAOsSCSb*) and two malate dehydrogenases (*Os05g0574400*, *FLO16*) in the TCA cycle were up-regulated, whereas succinyl dehydrogenase (*OsSDH2-2*) was down-regulated (Fig. 3 and Additional file 2: Table S2). And, compared with NIP, the *OsPHYB* mutants exhibited changes in the expression of 33 starch metabolic genes. For example, genes involved in the metabolism of sucrose, cellulose, and hemicellulose were significantly up-regulated, whereas the genes for sucrose (*SUS4*) and starch (*OsSSIIIa/Flo5*) synthesis were significantly down-regulated. In addition, *FLO2*, *FLO7* and *FLO11* were also significantly reduced in *osphyb* (Fig. 3 and Additional file 2: Table S2). The α/β-amylase genes (*OsAmy3E* and *Os09g0569200*) were up-regulated in *osphyb*, while the α/β-amylase repressor genes (*RAG1*, *RAG2*, and *RA5B*) were down-regulated (Fig. 3 and Additional file 2: Table S2). Moreover, *ISA1* was also found to be significantly up-regulated in *osphyb*, whereas *SSG4* was repressed (Fig. 3 and Additional file 2: Table S2). The results indicated that glycolytic activation, changes in the TCA cycle, and starch morphology and composition affected grain size and chalk formation.

**Fig. 4:**
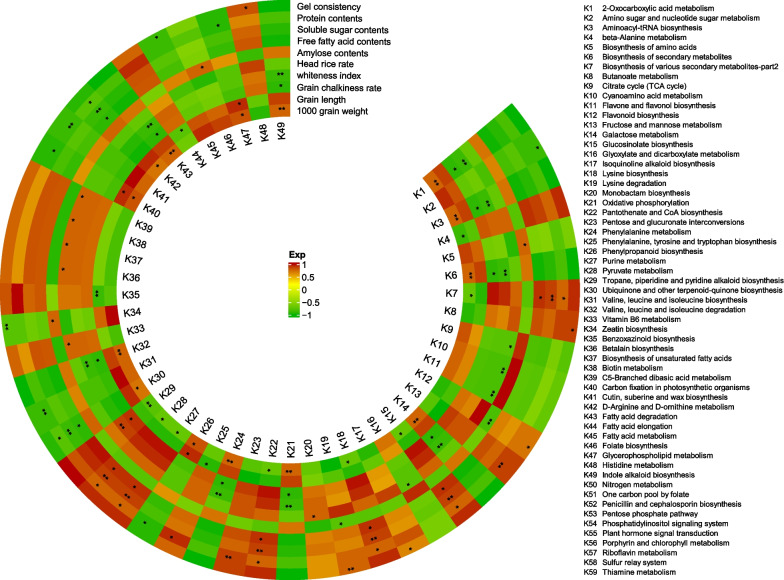
Metabolic pathway analysis related to grain size and quality in *osphyb*. Correlation coefficient are represented by a colour scale from green (negative correlation) to red (positive correlation). *P < 0.05, ** *P* < 0.01

Further analysis indicated that the genes for amino acid synthesis, such as *OsSAMS3* and *OsDHQS*, and those for amino acid metabolism, such as *OsMTK2*, *OsGDH2*, and *OsAdSS*, were significantly up-regulated in *osphyb*. Three amino acid transporter (*OsATL6*, *OsCAT6*, *OsCAT11*) and four storage protein genes (*Os02g0224300*, *Os02g0456100*, *OsEnS-51*, *Os06g0507150*) were down-regulated (Fig. 4 and Additional file 2: Table S2). Storage lipids are vital components to maintain the structure of seed storage substances and valuable for rice quality and food texture. The deletion of *OsPHYB* resulted in the significant up-regulation of all 6 differential fatty acid synthesis genes, as well as 11 lipid transporter and nine glyceride hydrolase genes compared with NIP (Fig. 3 and Additional file 2: Table S2). The results indicated that the *OsPHYB* mutation leads to changes in the level of expression of amino acids, storage proteins and fatty acids in grains, which could be one of the reasons for the increase in the number of grains and their chalkiness.

### OsPHYB Regulates the Expression of Genes Involved in Hormone Signaling

The deletion of *OsPHYB* altered the expression of 64 hormone signaling genes, including 10 genes related to auxin, four related to CK, 15 related to GA, 12 related to ABA, seven related to BRs, 16 related to ethylene (ETH) compared with the levels of expression in NIP (Fig. 3 and Additional file 2: Table S2). Among them, IAA (*OsIAA7*, *OsIAA11*, and *OsCLIP*), GA (*OsGAE1*, *OsGASR1*, *OsGASR9*, *OsGRAS32*, and *OsGIF2*), BR (*OsBZR1*, *OsBLE1*, and *OsGSK3*) and CK (*OsAHP1*) signaling genes in *osphyb* were all up-regulated, while the IAA efflux protein *OsPIN9* and the GA signaling inhibitor SLRL1 were down-regulated, indicating that the mutation in *OsPHYB* promoted the signaling and accumulation of IAA, GA, BR and CK. Further analysis found that the ABA synthesis gene *OsNCED1*, ABA receptor genes *OsPYL4* and *OsPYL5*, 11 ethylene-responsive factors (ERFs) and *OsEIL3* ETH signaling genes in *osphyb* were down-regulated, indicating that the *OsPHYB* mutation inhibited the synthesis of ABA and the signal transduction and accumulation of ETH. The results indicated that changes in the expression of phytohormones in grains caused by *OsPHYB* mutations are important regulators of *osphyb* grain gain and chalky formation.

### OsPHYB reGulates the Differential Expression of Oxidative Phosphorylation and Antioxidant Pathway Genes

A previous study found that *FLO16* was significantly up-regulated in the TCA cycle after the deletion of *OsPHYB*, whereas *OsSDH2-2* was down-regulated (Fig. 3 and Additional file 2: Table S2). *FLO16* has been reported to play a key role in redox homeostasis (Centeno et al. 2011; Teng et al. 2019). In addition, *OsSDH2-2* (succinate dehydrogenase) and the TCA cycle are one of the centers that links oxidative phosphorylation and electron transfer. Mitochondrial complex I and complex III are considered to be the major sites of ROS production in plant mitochondria. Therefore, the deletion of *OsPHYB* is bound to cause redox homeostasis in *osphyb*.

Compared with NIP, the *OsPHYB* mutants 15 differentially genes in the oxidative phosphorylation of complex I (NADH dehydrogenase), complex III (cytochrome c reductase/mitochondria), and complex IV (cytochrome c oxidase) all were significantly up-regulated, whereas the expression of complex II (succinate dehydrogenase) *OsSDH2-2* was down-regulated. In complex V (ATP synthase), the levels of expression of nine genes changed; six ATP synthase and *Chalk5* were significantly up-regulated, and two plasma membrane (PM) H^+^-ATPases (*Os02g0313900*, *OsA7*) were down-regulated (Fig. 3 and Additional file 2: Table S2). In *OsPHYB* mutants, complexes I, III, IV, and V were activated, which increased the accumulation of ATP, whereas the repression of *OsSDH2-2* interfered with the production of ROS. Since ROS are essential for redox homeostasis in plants, the mutant *OsPHYB* resulted in significantly higher levels of expression of the genes related to antioxidant enzymes, such as *OsAPXs*, *OsDHAR1*, *OsGPX3*, *OsGSTs*, *OsMDAR3*, *OsSOD1*, and *OsPRXs*, whereas the levels of expression of the oxygen burst genes *Osrboh7* and *Osrboh9* were significantly down-regulated (Fig. 3 and Additional file 2: Table S2).

### OsPHYB Regulates Transcription–Translation and Cell Cycle Related Gene Expression Differences

Compared with NIP, the *OsPHYB* mutants substantially activated the transcriptional and translational regulation of seven RNA polymerase genes, 146 ribosomal protein genes, five RNA polymerase genes, 15 ribosomal biosynthesis genes, 18 ER protein synthesis export genes, and 12 translation initiation factor (eIF) family genes that were significantly up-regulated (Fig. 3 and Additional file 2: Table S2). Further analysis revealed that deletion of *OsPHYB* up-regulated the expression of both cyclin (*OsCycB2*, *OsCycB2;2*, *OsCycD3;2*) and cyclin-dependent kinase (*OsCDKB1;1*, *OsCDKB2;1*) genes but significantly inhibited the expression of the cyclin-dependent kinase inhibitor gene *OsKRP6*. In addition, 17 F-box genes were differentially expressed, and 14 of them were down-regulated (Fig. 3 and Additional file 2: Table S2). The results indicated that the enhanced expression of transcription-translation and cell cycle genes could be related to grain size and chalk formation.

### Metabolic Pathways Contributed to Grain Size and Quality in osphyb

There are 59 metabolic pathways related to grain size and quality traits, and varying rice traits are regulated by different metabolic pathways (Fig. 4). Among them, ascorbic acid metabolism, fatty acid metabolism, amino acid metabolism, and phytohormone signal transduction among others were consistent with the results in Sect. 3.3. This further confirmed the importance of these metabolic pathways in the regulation of grain size and quality by *OsPHYB*. The correlation analysis between metabolic pathways and grain traits showed that 25 metabolic pathways affected rice yield, and 14 were positively correlated, whereas 11 were negatively correlated (Fig. 4). The chalkiness trait was affected by 10 metabolic pathways; one was positively correlated, and nine were negatively correlated (Fig. 4). Ten metabolic pathways significantly affected the processing quality (head rice rate); six were positively correlated, and one was negatively correlated (Fig. 4). Taste and nutritional quality were affected by 24 metabolic effects with 14 positively correlated and 10 negatively correlated (Fig. 4). The correlation between amylose and other nutritional qualities and metabolic pathways was the opposite; it was affected by a total of five metabolic pathways with three positively correlated and two negatively correlated (Fig. 4). The results indicated that amino acid metabolism, organic acid metabolism, fatty acid metabolism, alkaloid metabolism, purine metabolism, and phenylpropane and flavonoid metabolism played an important role in the regulation of grain size and quality by *OsPHYB* mutation.

### A Correlation Analysis of the Effect of Different Metabolites from osphyb on Grain Size and Quality

**Fig. 5:**
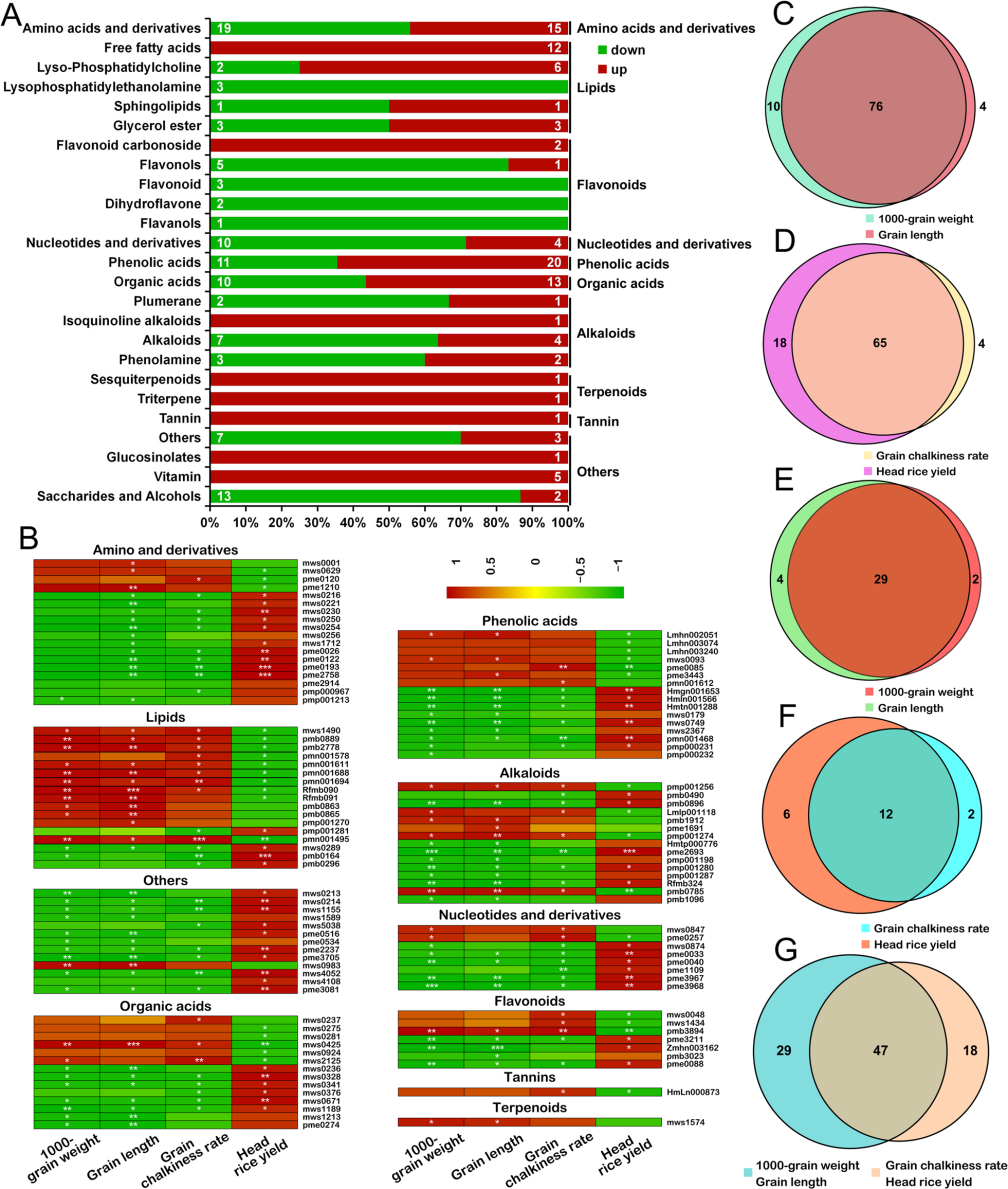
Correlation analysis of four grain traits and 201 different metabolites of *osphy*. **A** Analysis of the different metabolite types and increase and decrease levels between *osphyb* and NIP. **B** Analysis of 110 differential metabolites significantly related to *osphyb* 4 grain traits. Correlation coefficient are represented by a colour scale from green (negative correlation) to red (positive correlation). *P < 0.05, ** *P* < 0.01. **C** Analysis of metabolites significantly correlated with 1000 grain weight, grain length of *osphyb* among 201 differential metabolites. **D** Analysis of metabolites significantly correlated with grain chalkiness rate, head rice yield of *osphyb* among 201 differential metabolites. **E** Among the 201 differential metabolites, only the metabolites significantly correlated with the 1000 grain weight and grain length of *osphyb* were analyzed. **F** Among the 201 differential metabolites, only the metabolites significantly correlated with the grain chalkiness rate, head rice yield of *osphyb* were analyzed. **G** Metabolite analysis of 201 differential metabolites significantly correlated with *osphyb* 4 grain traits

Compared with NIP, the deletion of *OsPHYB* altered 201 significantly different metabolites that were detected in 10 categories, including amino acids and derivatives, flavonoids, phenolic acids, lipids, nucleotides, and derivatives, organic acids, alkaloids, and tannins. A total of 99 metabolites were significantly up-regulated, whereas 102 metabolites were down-regulated (Fig. 5A, Additional file 1: Fig. S6, Additional file 3: Table S3). Among them, the contents of FFA, lysophosphatidylcholine (LPC), vitamins, and most organic acids significantly increased. Saccharides and alcohols, flavonoids and nucleotides, and derivatives significantly decreased (Fig. 5A and Additional file 3: Table S3). Changes in these metabolic pathways were caused by changes in transcription, such as fatty acid synthesis, amino acid synthesis and metabolism, and the TCA cycle (Fig. 3, Additional file 2: Table S2). In addition, nucleotides, flavonoids, and organic acids among others were also regulated by their abundance of expression at the transcriptional level (Additional file 1: Fig. S7).

The correlation analysis of 201 differential metabolites and four grain traits in rice found that 110 metabolites were significantly associated with four grain traits, including the 1,000-grain weight, grain length, grain chalkiness rate, and head rice rate (*P* < 0.05) (Fig. 5B and Additional file 3: Table S3). An additional analysis with a Venn diagram indicated that 76 of the 110 metabolites significantly correlated with the yield traits of 1,000-grain weight and grain length (*P* < 0.05), whereas only 29 in 76 significantly correlated with the 1,000-grain weight and grain length (*P* < 0.05) (Fig. 5C, E). A total of 65 of the 110 metabolites significantly correlated with the rates of chalky grains and whole milled rice (*P* < 0.05), whereas 12 of the 65 only significantly correlated with the rates of chalky grains and whole milled rice (*P* < 0.05) (Fig. 5D, F). A total of 29 in the metabolic profile of 110 were significantly associated with all four grain traits (*P* < 0.05) (Fig. 5G).

Among them, the increase in all FFA and almost all LPC metabolites in the significantly related lipids significantly positively correlated with the 1,000-grain weight and grain length of *osphyb*, whereas the head rice rate negatively correlated. The increase in FFA positively correlated with the rate of grain chalkiness (Fig. 5B). The decrease of all the saccharides and alcohols that significantly correlated in others was almost negatively correlated with the 1,000-grain weight, grain length, and grain chalkiness rate of *osphyb*, whereas the head rice rate generally significantly positively correlated. All the amino acids and derivatives, organic acids, phenolic acids, alkaloids, nuclei and derivatives, and flavonoids that decreased were almost significantly negatively correlated with the 1,000-grain weight, grain length, and grain chalkiness rate but positively correlated with the head rice rate (Fig. 5B).

### Network Diagram and Correlation Analysis of Primary Metabolic Pathways

**Fig. 6:**
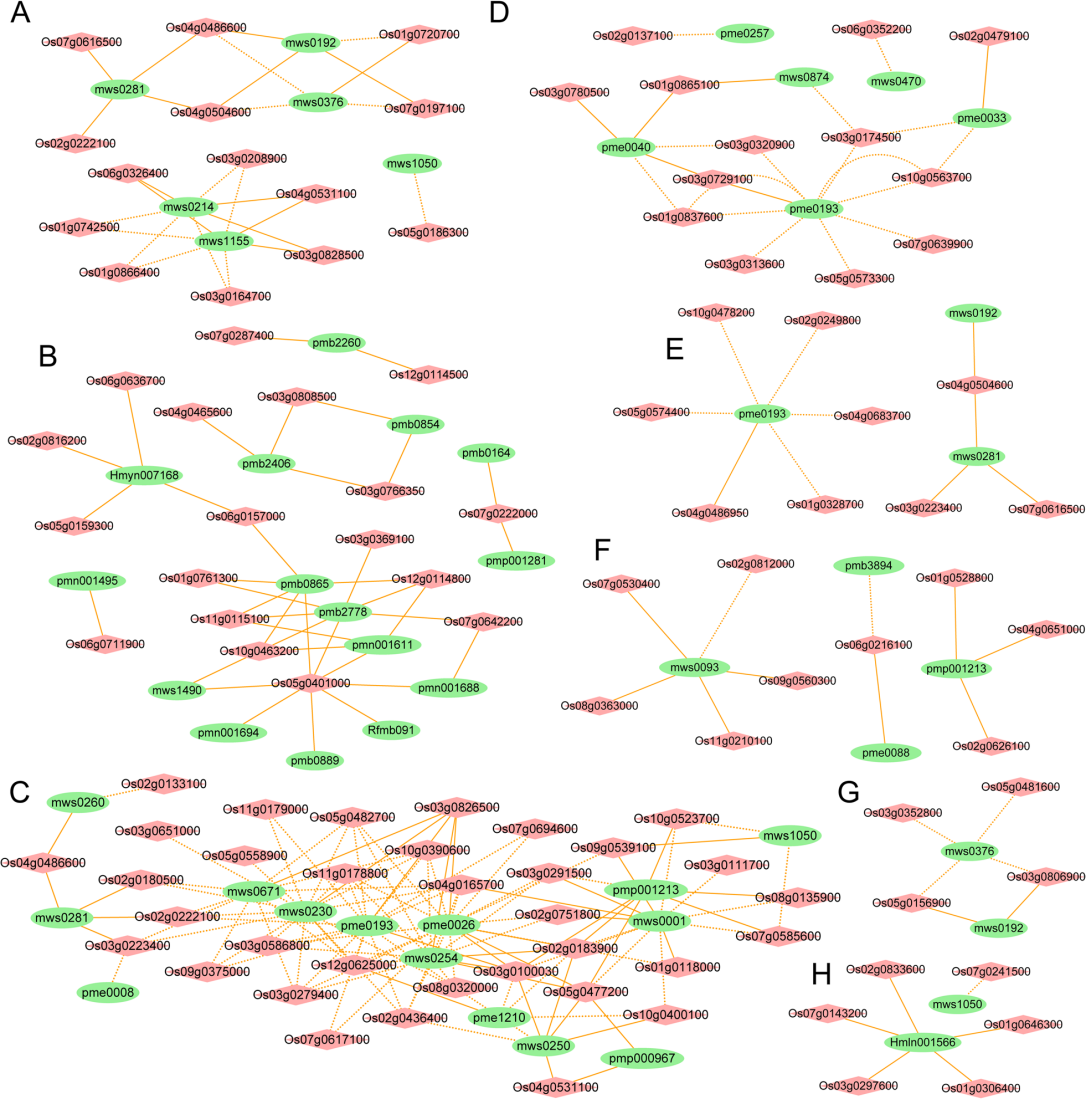
Metabolites and genes network of the eight primary metabolic pathways. **A**–**H** Carbon metabolism, lipid metabolism, amino acids and derivatives, nucleotides, organic acids, phenylpropane and flavonoids, oxidative phosphorylation and hormone signaling related pathway network set. Red diamonds represent differential genes. Green ovals represent differential metabolism. Solid orange line represents a positive correlation. Orange dotted line represents a negative correlation. PCC > 0.9, PCC-*P* < 0.01

A Pearson's correlation coefficient analysis was conducted based on the metabolome and transcriptome profiles and resulted in a Pearson correlation coefficient (PCC) > 0.9, PCC-*P* < 0.01). The screening results showed that 42 metabolites significantly correlated with 101 genes (Fig. 6, Additional file 4: Table S4). Eight pathway network sets related to carbon metabolism, lipid metabolism, amino acids and derivatives, nucleotides, organic acids, phenylpropane and flavonoids, oxidative phosphorylation and hormone signaling were obtained (Fig. 6). As more genes or metabolites connected, their range of influence became larger. In the carbon metabolism pathway, only mws0281 (citrate) positively correlated with these pathway genes (Fig. 6A). In the lipid metabolism pathway, all 15 metabolites positively correlated with genes (Fig. 6B). In the amino acids and derivatives pathway, three out of 14 metabolites (mws0281, pmp000967, pmp001213) positively correlated, and the rest, such as mws0001, mws0230, mws0254, mws0671, pme0026, pme0193, negatively correlated (Fig. 6C). In the nucleotide, organic acid, phenylpropanoid, and flavonoid pathways, six, three, and four metabolites were associated with these pathway genes, respectively (Fig. 6D–F). In the oxidative phosphorylation pathway, mws0376 (fumaric acid) negatively correlated with these pathway genes, whereas mws0192 (succinic acid) positively correlated (Fig. 6G). In the hormone signaling pathway, mws1050 (O-acetylserine) negatively correlated with these pathway genes, while Hmln001566 (salicylic acid) positively correlated (Fig. 6H).

## Discussion

Increasing grain yield and improving grain quality are two important goals of rice breeding. Particle size is not only a yield trait but also an important appearance quality of rice, and chalkiness is one of the most important indicators of grain quality (Jiang et al. 2022; Yang et al. 2022). In this study, *OsPHYB* mutations increased the kernel size and chalkiness, while altering the composition of kernel nutrients. Furthermore, these alterations were shown to be associated with the physiological, transcriptomic, and metabolomic changes caused by *OsPHYB* mutations. Therefore, our findings may contribute to future molecular breeding of rice that focuses on improving grain yield and quality.

### OsPHYB Plays a Key Role in the Regulation of Grain Quality

Chalkiness is closely related to kernel size, often negatively correlating (Miyazaki et al. 2018). This is consistent with our findings that the deletion of *OsPHYB* resulted in increased grain size and chalkiness (Fig. 1). Kernel size is determined by the endosperm cell number and area, while chalkiness is closely related to abnormal endosperm cell development (Morita et al. 2005), uneven accumulation of starch, and shape (Ji et al. 2019; Lei et al. 2022). Changes in the contents of amylose, fatty acids, and soluble sugar can alter the quality of rice grains (Dong et al. 2014; Nakata et al. 2017; Wang et al. 2015a). These results were consistent with the results that *OsPHYB* mutation resulted in significant changes in the number of grain cells, the morphology of complex starch granules, and the content of nutrients (Fig. 2; Table 1). Taken together, the knockout of *OsPHYB* increased the contents of FFA, soluble sugars, amylose metabolites, and promoted the assimilation of material, leading to increased grain size and a higher 1,000-grain weight, but the rapid enlargement of grains could result in loosely packed starch granules and thus, a highly chalky appearance in *osphyb*.

### OsPHYB Could Affect Grain Quality Through Carbon Metabolism Genes

Previous research reported that carbon metabolism plays vital roles in starch synthesis and the formation of chalkiness (Liu et al. 2010). As an end product of glycolysis and a key substrate of mitochondrial respiration, pyruvate plays a pivotal role in plant carbon metabolism (O’Leary 2021). The *OsPPDKB/FLO4* mutation increases the contents of amylose and lipids in the endosperm and causes a powdery endosperm, while increasing the supply of pyruvate for lipid synthesis. (Lappe et al. 2018; Wang et al. 2020). The overexpression of *FLO16* significantly increased the grain size and weight (Teng et al. 2019), whereas mutations in *FLO2*, *OsSSIIIa/Flo5*, and *FLO7* have been reported to cause changes in starch-related traits in grains, resulting in silty endosperm (Ryoo et al. 2007; She et al. 2010; Zhang et al. 2016). The changes in levels of expression of ISA1, SSG4, and RAG2 and the sucrose, cellulose, hemicellulose, and amylase genes were all key triggers of grain size and chalkiness in grain development (Hakata et al. 2012; Matsushima et al. 2014; Peng et al. 2014b; Zhou et al. 2017). The changes of expression of these genes were consistent with the results of this study (Table 1, Figs. 2G-I and 3).

Multiple genes involved in protein metabolism have been reported to mediate chalky formation (Xie et al. 2021), and multiple amino acid transporters can improve the quality of rice by regulating the contents of amino acids and storage proteins (Guo et al. 2020; Ji et al. 2020; Lin et al. 2017a; Lu et al. 2018). Thus, changes in the abundance of the expression of amino acid metabolism and the repression of expression of storage protein genes led to the increased chalkiness of *osphyb*. Fatty acids have also been reported to be key factors that affect grain size, yield and quality (Guo et al. 2013; Sandoval 2012; Wang et al. 2015a; Zhao et al. 2019). Therefore, the differential expression of these genes resulted in changes in protein and FFA content in *osphyb* grains (Table 1, Fig. 3). Lipids are closely related to starch synthesis, and changes in the biosynthesis of lipids can significantly affect the changes in starch content (Cai et al. 2018; Xi et al. 2020). In conclusion, the mutation of *OsPHYB* mediated the levels of expression of the genes in carbon metabolic pathways, the coordinated regulation of carbon allocation by these dynamic regulatory processes resulted in changes in grain size and the formation of chalkiness in *osphyb*.

### OsPHYB Could Affect Grain Quality Through Phytohormone Signaling Genes

Plant hormones are important regulators of grain size and chalkiness formation in rice (Sou et al. 2006; Xie et al. 2021). This study indicated that *OsPHYB* mutations activated IAA, GA, CK, and BR signaling and accumulation, while ABA and ETH synthesis and/or signaling were inhibited (Fig. 3). This is consistent with previous studies that reported that IAA, GA, CK, BR and ABA regulate grain shape and chalkiness (Jiang et al. 2022; Xiao et al. 2017; Zhang et al. 2020).

*BG1* is involved in the regulation of auxin transport and response, and the overexpression of *BG1* improves grain size and weight (Liu et al. 2015). *OsIAA7*, *OsIAA11,* and *OsCLIP* in *osphyb* were up-regulated, whereas *OsPIN9* was downregulated (Fig. 3). The overexpression of *OsGASR9* and *OsBZR1* increased grain size and weight (Li et al. 2019; Zhu et al. 2015), whereas the inhibition of ETH could enhance the level of expression of starch synthase and cell cycle genes and lead to increased grain size (Panda et al. 2016). In addition, high levels of ethylene and 1-aminocyclopropane-1-carboxylate (ACC) in grain inhibited cell division of the endosperm, resulting in decreased grain weight and increased chalkiness (Zhang et al. 2009). The overexpression of *OsPYL/RCAR5* severely reduced grain yield, whereas *OsPYL/RCAR5* was significantly inhibited in *osphyb* (Kim et al. 2014). The results of this study are consistent with those of previous studies (Fig. 3). *OsAO3* is involved in the biosynthesis of ABA and negatively regulates the yield of rice grains (Shi et al. 2021). The ABA synthesis gene *OsNCED1* in *osphyb* was significantly down-regulated, and the ABA receptor gene was also significantly down-regulated (Fig. 3). In conclusion, phytohormones play an important role in grain size and chalkiness during endosperm development in rice. The differential expression of genes in phytohormonal signaling also results in changes in the expression of responsive genes, which could be an additional reason for grain size and chalkiness formation.

### OsPHYB Could Affect Grain Quality by Promoting ATP Production, Regulating pH Homeostasis and Enhancing Oxidative Tolerance

Recent studies have identified a close correlation between redox homeostasis and the development and chalkiness of rice grains (Liu et al. 2010; Suriyasak et al. 2017; Xu et al. 2010), suggesting that ROS may play a critical role in the regulation of rice endosperm chalkiness (Liu et al. 2010; Xu et al. 2010). Existing studies have shown that the overexpression of *Chalk5* can perturb the pH homeostasis of the endosperm membrane transport system, thereby affecting proteosome biogenesis and increasing the chalkiness of endosperms (Li et al. 2014). The PM H^+^-ATPases are also involved in cellular growth, sugar transport, mineral nutrient translocation, and grain filling among others and are involved in cell growth by reducing the apoplast pH and activating the expansins responsible for cell expansion (Falhof et al. 2016; Gaxiola et al. 2007). This was consistent with changes in the expression of *Chalk5* and PM H^+^-ATPase (*OsA7*, *Os02g0313900*) in *osphyb* (Fig. 3). This study also indicated that the mutation of *OsPHYB* can also activate the expression of oxidative phosphorylation complexes I, III, and IV to promote the production of ATP (Fig. 3).

Ascorbic acid deficiency leads to the accumulation of hydrogen peroxide (H_2_O_2_), which affects antioxidant capacity and photosynthetic function, alters enzyme activities and gene transcript abundances related to starch synthesis, and ultimately leads to increased grain chalkiness (Yu et al. 2017), while its increased content enhanced the tolerance of oxidatively stressed grains and reduced chalkiness (Lin et al. 2022). ROS can lead to rice grain chalkiness by pathways that overlap with seed germination (Suriyasak et al. 2017). In this study, the levels of expression of antioxidant enzyme-related genes, such as *OsAPXs*, *OsDHAR1*, *OsGPX3*, *OsGSTs*, *OsMDAR3*, *OsSOD1*, and *OsPRXs*, in *osphyb* increased significantly, while the expression of oxygen burst genes (*Osrboh7* and *Osrboh9*) was down-regulated (Fig. 3, Additional file 2: Table S2). Thus, the increase in *osphyb* grain and chalkiness could be regulated by activating oxidative phosphorylation, enhancing oxidative tolerance, and disrupting pH homeostasis.

### OsPHYB Could Affect Grain Quality by Activating Transcription-Translation and Cell Cycle Genes

Ribosomes are the cellular machinery that performs protein synthesis by translating the information contained in mRNA molecules (Hammerling et al. 2020). RNA polymerase activity plays an important role in rice development and yield-related traits (Jha et al. 2021; Zhao et al. 2020). *GL6* interacts with *RPC53* and *TFC1*, participates in the RNA polymerase III transcription machinery, and regulates the expression of genes involved in rice grain development (Wang et al. 2019). Ribosomal gene mutations primarily manifest as a variety of developmental defects, such as *OsRPL3B* and *OsRPS3A* (Byrne 2009; Uzair et al. 2021; Zheng et al. 2016). In this study, the mutation of *OsPHYB* activated the expression of a large number of RNA polymerase and ribosomal genes (Fig. 3). Translational regulation is ubiquitous in cellular processes, and the cell cycle is a highly orchestrated process that is extensively regulated to ensure accurate DNA replication and proper chromosome segregation (Stumpf et al. 2013). *OsmiR396a*/*OsGRFs* have been reported to act between SLR1 and the cell cycle-related genes *OsCycB2* and *OsCycB2;2* to mediate the proliferation of rice cells (Lu et al. 2020). The expression of *OsCycB1;1* is critical for endosperm formation via the regulation of mitotic division, and the endosperm plays an important role in the maintenance of embryo development in rice, whereas *OsCYCD3;1* promotes branch formation (Guo et al. 2010; Ohyama et al. 2022). The levels of expression of *OsKRP1* and *OsKRP2* play an important role in rice endosperm development, grain filling, and cell proliferation (Ajadi et al. 2019). Therefore, the grain enlargement and chalky formation of *osphyb* could be owing to the regulatory role of RNA polymerase, ribosome and cell cycle pathways in growth and development, which are achieved by regulating translation and cell cycle.

### OsPHYB Mutation Alters Grain Metabolic Pathways and the Content of Metabolites, Such as FFA and LPC Among Others

It has been reported that the formation of chalky endosperm is related to a reduction in the metabolites related to carbon and nitrogen metabolism that are involved in starch storage and protein synthesis, whereas the alkaloids, phenolic acids, amino acids, flavonoids, and starch metabolism are all closely related to the biosynthesis of proteins and the accumulation of starch in chalky endosperm (Chen et al. 2020; Kim et al. 2013; Lin et al. 2017b). Starch lipids are specific to cereal endosperm starch and are primarily composed of FFA and LPC; these fatty acids strongly influence the assembly and properties of cereal starch (Gayral et al. 2015). *OsACOT* is a key enzyme in fatty acid desaturation and elongation, particularly the transformation of palmitic acid (16:0) to linoleic acid (18:2), and miR1432-*OsACOT* regulates the increase in particle size and grain filling by regulating the biosynthesis of long-chain fatty acids (Zhao et al. 2019). Therefore, the increases in FFA and LPC caused by the mutation in *OsPHYB* could affect the development and structure of amyloplasts and starch granules in grains, resulting in larger kernels and increased chalkiness in *osphyb*.

The increase in content of soluble sugar in high chalky grains comes at the expense of starch degradation (Nakata et al. 2017). The decrease of saccharides and alcohols in *osphyb* could be caused by the changes in related metabolic enzymes, such as amylase, at the transcriptional level. Aspartate aminotransferase and amino acid permease can regulate the contents of rice grain proteins, nutritional quality, and yield (Lu et al. 2012; Peng et al. 2014a). Adjusting the concentration of amino acids can promote bud growth and increase the number of tillers, which could improve grain yield (Lu et al. 2018). Almost all the decreased metabolism of amino acids and derivatives in *osphyb* were significantly associated with increased chalkiness, which could be caused by the effect of changes in the composition of amino acids on grain development. Maltooligosaccharides, linear glucans, monosaccharides, and organic acids are involved in starch biosynthesis in rice endosperm (Nakamura et al. 2020). Fumarase catalyzes the reversible hydration reaction between fumarate and L-malate in the TCA cycle. Therefore, the organic acids that are significantly related to grain and chalkiness in *osphyb* were significantly reduced, whereas these changes in organic acids could affect the synthesis of starch during grain development, whereas the reduction in fumaric acid affected the normal metabolism of the TCA cycle. The phenolic ring can stabilize and delocalize unpaired electrons, conferring an antioxidant property to phenolic acids. Phenolic acids are considered to be natural antioxidants that can scavenge the free radicals that could increase oxidative stress and potentially damage the large biological molecules, such as lipids, proteins, and nucleic acids (Adom and Liu 2002; Ratseewo et al. 2019). Therefore, the content of phenolic acids positively correlated with the antioxidant capacity of rice (Yu et al. 2021b). Flavonoids constitute a major group of plant phenolic compounds. Rice flavonoids primarily have antioxidant properties, although some of them have not been evaluated for their antioxidant activities (Cho et al. 2013; Goufo and Trindade 2014). *OsCOP1* regulates flavonoid biosynthesis and embryogenesis in rice seeds, thereby regulating the development of pericarp and embryos (Kim et al. 2021). The reduction in organic acids, phenolic acids, and flavonoids significantly related to grain traits in *osphyb* could be related to the enhanced ROS scavenging activated by the ROS antioxidant capacity of *osphyb*. Purine bases and nucleosides are produced by the turnover of nucleotides and nucleic acids, as well as from some cellular metabolic pathways (Ashihara et al. 2018). The decrease in adenine, guanine, and other nucleotides and derivatives significantly related to their grain traits in *osphyb* could be the result of *OsPHYB* mutations on its genetic level of metabolism and a series of related cellular metabolic pathways. A network analysis of the metabolites and genes of eight major metabolic pathways reaffirmed these results, particularly the lipid metabolism pathways. In conclusion, the increase of grain size and chalkiness caused by the deletion of *OsPHYB* was regulated by various metabolic pathways and metabolites, which ultimately led to changes in the composition of endosperm and the development and accumulation of starch.

## Conclusions

**Fig. 7:**
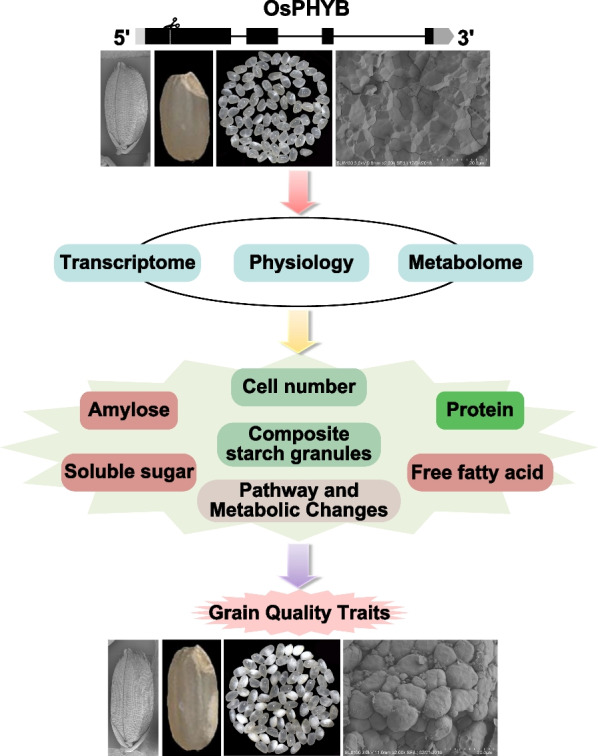
Schematic model of the regulatory network of *OsPHYB* mutation regulating grain size and chalkiness formation

A mutation in *OsPHYB* increased grain size and chalky formation in rice, which are complex traits controlled by multiple genes and metabolic pathways. The transcriptomic and metabolomic analyses showed that the induction in grain enlargement and the formation of chalkiness that resulted from the deletion of *OsPHYB* were complex and owing to a multi-factor coordinated network regulation (Fig. 7). In particular, a variety of key carbon metabolic genes, starch synthesis, and starch grain development genes, plant hormone genes, cell cycle genes, transcriptional regulation of oxidative phosphorylation and fatty acid synthesis pathways, and the increase in free fatty acid metabolites are critical for the grain size and chalk formation regulated by *OsPHYB*. Among them, the repressed expression of *OsPPDKA*, *OSSSIIA/Flo5*, *Flo2*, *Flo7*, *SSG4*, *RAG2* genes and the up-regulated expression of *Chalk5* gene are extremely important for *OsPHYB*-involved rice quality regulation. The up-regulation of *OsBZR1*, *OsGASR9*, *OsCycB2*, *OsCycB2;2*, *OsCycD3;2*, *OsCDKB1;1*, *OsCDKB2;1* genes which induced by *OsPHYB* mutation positively contributed to grain size increase of *osphyb*. Taken together, these results revealed new functions of *OsPHYB* in rice grain development and provide a new strategy for yield and quality improvement in rice breeding.

## Materials and Methods

### Plant Materials and Growth Conditions

The CRISPR/Cas9 expression vector pBUN411 used to create the *osphyb* mutation was provided by Prof. Qijun Chen, China Agricultural University (Beijing, China) (Xing et al. 2014). A 19 bp sequence (5'-TCCTCCACCGCATCGATGT-3') was selected as the target site before the protospacer adjacent motif (PAM) sequence (CGG) near the N-terminal of the first exon of *OsPHYB*. Nipponbare (NIP) was the variety of *japonica* rice that was transformed.

All the rice plants used in this study were grown naturally in 2019–2020 during the growing season and maintained at the Henan Agricultural University, Zhengzhou (E 112°42′–114°14′, N 34°16′–34°58′), Henan Province, China. The planting density was 13 × 30 cm, with one plant per hill.

### Analysis of Rice Grain Phenotypes

The harvested grains were air-dried at room temperature and stored for 3 months. Thirty plump grains were randomly selected from transgenic and wild-type plants, and their grain length, width, and thickness were measured using a Vernier caliper. The weight of 1000 grains was determined using a granulometer (CanoScan 5600F; Canon, USA) and electronic balance. The rate of chalkiness was measured using a rice appearance quality detector (JMWT12; Dongfu JiuHeng, Beijing, China). At least five biological replicates were analyzed.

### Scanning Electron Microscopy Analysis (SEM)

They were observed with a SEM (SU8100; Hitachi, Tokyo, Japan) after gold was sprayed on the natural fracture cross-section of rice grains and polished rice. They were then photographed with an accelerating voltage of 3.0 kV. All the procedures were conducted according the manufacturer’s instructions.

### Gene Expression Analysis

Total RNA was extracted using the TRIzol reagent (Invitrogen, Carlsbad, CA, USA) according to the manufacturer’s instructions, and then reverse transcription was performed using reverse transcriptase (Promega, Madison, WI, USA). qRT-PCR (real-time quantitative reverse transcription PCR) was performed using GoTaq, a CFX 96 real-time system qRT-PCR system (Bio-Rad, Hercules, CA, USA) according to the manufacturer’s instructions for the quantitative PCR master mix (Promega). The gene-specific primers are listed in Additional file 5: Table S1. The following conditions were used for the PCR: initial denaturation at 95 °C for 3 min, then 40 cycles of 15 s at 95 °C, 30 s at 58 °C, and 30 s at 72 °C. The relative level of expression was calculated using the 2^−ΔΔCT^ method (Livak and Schmittgen 2001). The primers for transcriptome qRT-PCR verification are shown in Additional file 5: Table S1.

### Transcriptomic Analysis

RNA-seq was performed using RNA that was extracted from the endosperm samples of wild-type and *OsPHYB* knockout plants 12 days after flowering (DAF) using the TRIzol reagent. The purity and quality of the total RNA were checked according to the manufacturer's instructions. Qualified and high-quality total RNA samples were reverse transcribed into cDNA used to construct the cDNA library. An Illumina HiSeq high-throughput sequencing platform (Illumina, San Diego, CA, USA) was used to sequence the cDNA library based on sequencing by synthesis (SBS) technology. Three biological replicate samples were prepared for NIP and the mutant plants. After sequencing, the data were analyzed using the rice genome sequence (https://rapdb.dna.affrc.go.jp/download/irgsp1.html) as a reference. Differential expression analysis, gene ontology (GO) and Kyoto Encyclopedia of Genes and Genomes (KEGG) pathway enrichment analyses and quantitative qRT-PCR analysis were conducted as previously described (Zhu et al. 2018).

### Metabolite Profiling and Data Analysis

The metabolites extracted from the rice 12 DAF grains sampled were measured using UPLC-MS/MS technology combined with data-dependent acquisition (Chen et al. 2013). XCMS software was used for peak extraction and metabolite identification, and multidimensional statistical analysis of the mass spectrometry data was conducted using SIMCA software (Fraga et al. 2010). An orthogonal partial least squares discriminant analysis (OPLS-DA) was used to filter out noises (Thevenot et al. 2015). The UPLC conditions are shown in the Additional file 1.

### Widely Targeted Metabolomics Assay

A correlation analysis was performed between the differential metabolite data and rice grain traits, and the Pearson correlation coefficient was calculated using R (Foundation for Statistical Computing, Vienna, Austria). The correlation heatmap was visualized using the *pheatmap* package in R. A Venn diagram was plotted using the *VennDiagram* package of R to visualize the significantly correlated genes.

## Supplementary Information

**Additional file 1.** Supplementary Materials and Figures.

**Additional file 2. Table S2**. Gene names and numbers for differential pathways.

**Additional file 3. Table S3**. The name and number of metabolites.

**Additional file 4. Table S4**. Network statistics of differential metabolites and related genes.

**Additional file 5. Table S1**. List of primers required for this study.

## Data Availability

The datasets supporting the conclusions of this article are included within the article and its additional files.

## Abbreviations

ABA: Abscisic acid
ACC: 1-Aminocyclopropane-1-carboxylate
BRs: Brassinosteroids
CKs: Cytokinins
DAF: Days after flowering
DEG: Differentially expressed gene
ERF: Ethylene-responsive
ETH: Ethylene
FFA: Free fatty acids
FPKM: Fragments per kilobase of transcript per million mapped fragments
GAs: Gibberellins
GO: Gene ontology
KEGG: Kyoto Encyclopedia of Genes and Genomes
LPC: Lysophosphatidylcholine
NIP: Nipponbare
*OsPHYB*: *Oryza sativa* *phytochrome B*
OPLS-DA: Orthogonal partial least squares discriminant analysis
PCC: Pearson correlation coefficient
PM: Plasma membrane
PPDK: Pyruvate phosphate dikinase gene
qRT-PCR: Real-time quantitative reverse transcription PCR
ROS: Reactive oxygen species
SBS: Sequencing by synthesis
SEM: Scanning electron microscope
TCA: Tricarboxylic acid

## References

[CR1] Adom KK, Liu RH (2002). Antioxidant activity of grains. J Agric Food Chem.

[CR2] Ajadi AA, Tong X, Wang H, Zhao J, Tang L, Li Z, Liu X, Shu Y, Li S, Wang S (2019). Cyclin-dependent kinase inhibitors KRP1 and KRP2 are involved in grain filling and seed germination in rice (*Oryza sativa* L.). Int J Mol Sci.

[CR3] Ashihara H, Stasolla C, Fujimura T, Crozier A (2018). Purine salvage in plants. Phytochemistry.

[CR4] Byrne ME (2009). A role for the ribosome in development. Trends Plant Sci.

[CR5] Cai Y, Zhang W, Jin J, Yang X, You X, Yan H, Wang L, Chen J, Xu J, Chen W (2018). *OsPKpalpha1* encodes a plastidic pyruvate kinase that affects starch biosynthesis in the rice endosperm. J Integr Plant Biol.

[CR6] Centeno DC, Osorio S, Nunes-Nesi A, Bertolo AL, Carneiro RT, Araujo WL, Steinhauser MC, Michalska J, Rohrmann J, Geigenberger P (2011). Malate plays a crucial role in starch metabolism, ripening, and soluble solid content of tomato fruit and affects postharvest softening. Plant Cell.

[CR7] Chen W, Gong L, Guo Z, Wang W, Zhang H, Liu X, Yu S, Xiong L, Luo J (2013). A novel integrated method for large-scale detection, identification, and quantification of widely targeted metabolites: application in the study of rice metabolomics. Mol Plant.

[CR8] Chen Z, Chen H, Jiang Y, Wang J, Khan A, Li P, Cao C (2020). Metabolomic analysis reveals metabolites and pathways involved in grain quality traits of high-quality rice cultivars under a dry cultivation system. Food Chem.

[CR9] Cho JG, Song NY, Nam TG, Shrestha S, Park HJ, Lyu HN, Kim DO, Lee G, Woo YM, Jeong TS, Baek NI (2013). Flavonoids from the grains of C1/R-S transgenic rice, the transgenic *Oryza sativa* spp. japonica, and their radical scavenging activities. J Agric Food Chem.

[CR10] Dong M, Gu J, Zhang L, Chen P, Liu T, Deng J, Lu H, Han L, Zhao B (2014). Comparative proteomics analysis of superior and inferior spikelets in hybrid rice during grain filling and response of inferior spikelets to drought stress using isobaric tags for relative and absolute quantification. J Proteom.

[CR11] Falhof J, Pedersen JT, Fuglsang AT, Palmgren M (2016). Plasma membrane H^(+)^-ATPase regulation in the center of plant physiology. Mol Plant.

[CR12] Ferreira CD, Lang GH, Lindemann IDS, Timm NDS, Hoffmann JF, Ziegler V, de Oliveira M (2021). Postharvest UV-C irradiation for fungal control and reduction of mycotoxins in brown, black, and red rice during long-term storage. Food Chem.

[CR13] Fraga CG, Clowers BH, Moore RJ, Zink EM (2010). Signature-discovery approach for sample matching of a nerve-agent precursor using liquid chromatography-mass spectrometry, XCMS, and chemometrics. Anal Chem.

[CR14] Garg AK, Sawers RJ, Wang H, Kim JK, Walker JM, Brutnell TP, Parthasarathy MV, Vierstra RD, Wu RJ (2006). Light-regulated overexpression of an *Arabidopsis* phytochrome A gene in rice alters plant architecture and increases grain yield. Planta.

[CR15] Gaxiola RA, Palmgren MG, Schumacher K (2007). Plant proton pumps. FEBS Lett.

[CR16] Gayral M, Bakan B, Dalgalarrondo M, Elmorjani K, Delluc C, Brunet S, Linossier L, Morel MH, Marion D (2015). Lipid partitioning in maize (*Zea mays* L.) endosperm highlights relationships among starch lipids, amylose, and vitreousness. J Agric Food Chem.

[CR17] Goufo P, Trindade H (2014). Rice antioxidants: phenolic acids, flavonoids, anthocyanins, proanthocyanidins, tocopherols, tocotrienols, gamma-oryzanol, and phytic acid. Food Sci Nutr.

[CR18] Guo C, Ge X, Ma H (2013). The rice *OsDIL* gene plays a role in drought tolerance at vegetative and reproductive stages. Plant Mol Biol.

[CR19] Guo J, Wang F, Song J, Sun W, Zhang XS (2010). The expression of *Orysa;CycB1;1* is essential for endosperm formation and causes embryo enlargement in rice. Planta.

[CR20] Guo N, Gu M, Hu J, Qu H, Xu G (2020). Rice *OsLHT1* functions in leaf-to-panicle nitrogen allocation for grain yield and quality. Front Plant Sci.

[CR21] Hakata M, Kuroda M, Miyashita T, Yamaguchi T, Kojima M, Sakakibara H, Mitsui T, Yamakawa H (2012). Suppression of alpha-amylase genes improves quality of rice grain ripened under high temperature. Plant Biotechnol J.

[CR22] Hammerling MJ, Fritz BR, Yoesep DJ, Kim DS, Carlson ED, Jewett MC (2020). In vitro ribosome synthesis and evolution through ribosome display. Nat Commun.

[CR23] Hoshikawa, B.K. (1989). The growing rice plant: an anatomical monograph. Nosan Gyoson Bunka.

[CR24] Hu L, Tu B, Yang W, Yuan H, Li J, Guo L, Zheng L, Chen W, Zhu X, Wang Y (2020). Mitochondria-associated pyruvate kinase complexes regulate grain filling in rice. Plant Physiol.

[CR25] Jha V, Narjala A, Basu D, Sujith TN, Pachamuthu K, Chenna S, Nair A, Shivaprasad PV (2021). Essential role of gamma-clade RNA-dependent RNA polymerases in rice development and yield-related traits is linked to their atypical polymerase activities regulating specific genomic regions. New Phytol.

[CR26] Ji X, Du Y, Li F, Sun H, Zhang J, Li J, Peng T, Xin Z, Zhao Q (2019). The basic helix-loop-helix transcription factor, OsPIL15, regulates grain size via directly targeting a purine permease gene *OsPUP7* in rice. Plant Biotechnol J.

[CR27] Ji Y, Huang W, Wu B, Fang Z, Wang X (2020). The amino acid transporter AAP1 mediates growth and grain yield by regulating neutral amino acid uptake and reallocation in *Oryza sativa*. J Exp Bot.

[CR28] Jiang H, Zhang A, Liu X, Chen J (2022). Grain size associated genes and the molecular regulatory mechanism in rice. Int J Mol Sci.

[CR29] Kim B, Piao R, Lee G, Koh E, Lee Y, Woo S, Jiang W, Septiningsih EM, Thomson MJ, Koh HJ (2021). *OsCOP1* regulates embryo development and flavonoid biosynthesis in rice (*Oryza sativa* L.). Theor Appl Genet.

[CR30] Kim H, Lee K, Hwang H, Bhatnagar N, Kim DY, Yoon IS, Byun MO, Kim ST, Jung KH, Kim BG (2014). Overexpression of *PYL5* in rice enhances drought tolerance, inhibits growth, and modulates gene expression. J Exp Bot.

[CR31] Kim JK, Park SY, Lim SH, Yeo Y, Cho HS, Ha SH (2013). Comparative metabolic profiling of pigmented rice (*Oryza sativa* L.) cultivars reveals primary metabolites are correlated with secondary metabolites. J Cereal Sci.

[CR32] Lappe RR, Baier JW, Boehlein SK, Huffman R, Lin Q, Wattebled F, Settles AM, Hannah LC, Borisjuk L, Rolletschek H (2018). Functions of maize genes encoding pyruvate phosphate dikinase in developing endosperm. Proc Natl Acad Sci.

[CR33] Lei J, Teng X, Wang Y, Jiang X, Zhao H, Zheng X, Ren Y, Dong H, Wang Y, Duan E (2022). Plastidic pyruvate dehydrogenase complex E1 component subunit Alpha1 is involved in galactolipid biosynthesis required for amyloplast development in rice. Plant Biotechnol J.

[CR34] Li X, Shi S, Tao Q, Tao Y, Miao J, Peng X, Li C, Yang Z, Zhou Y, Liang G (2019). *OsGASR9* positively regulates grain size and yield in rice (*Oryza sativa*). Plant Sci.

[CR35] Li Y, Fan C, Xing Y, Yun P, Luo L, Yan B, Peng B, Xie W, Wang G, Li X (2014). *Chalk5* encodes a vacuolar H^(+)^-translocating pyrophosphatase influencing grain chalkiness in rice. Nat Genet.

[CR36] Lin CJ, Li CY, Lin SK, Yang FH, Huang JJ, Liu YH, Lur HS (2010). Influence of high temperature during grain filling on the accumulation of storage proteins and grain quality in rice (*Oryza sativa* L.). J Agric Food Chem.

[CR37] Lin F, Rensing C, Pang Z, Zou J, Lin S, Letuma P, Zhang Z, Lin W (2022). Metabolomic analysis reveals differential metabolites and pathways involved in grain chalkiness improvement under rice ratooning. Field Crop Res.

[CR38] Lin Z, Wang Z, Zhang X, Liu Z, Li G, Wang S, Ding Y (2017). Complementary proteome and transcriptome profiling in developing grains of a notched-belly rice mutant reveals key pathways involved in chalkiness formation. Plant Cell Physiol.

[CR39] Lin Z, Zhang X, Wang Z, Jiang Y, Liu Z, Alexander D, Li G, Wang S, Ding Y (2017). Metabolomic analysis of pathways related to rice grain chalkiness by a notched-belly mutant with high occurrence of white-belly grains. BMC Plant Biol.

[CR40] Liu L, Tong H, Xiao Y, Che R, Xu F, Hu B, Liang C, Chu J, Li J, Chu C (2015). Activation of *Big Grain1* significantly improves grain size by regulating auxin transport in rice. Proc Natl Acad Sci.

[CR41] Liu Q, Han R, Wu K, Zhang J, Ye Y, Wang S, Chen J, Pan Y, Li Q, Xu X (2018). G-protein betagamma subunits determine grain size through interaction with MADS-domain transcription factors in rice. Nat Commun.

[CR42] Liu X, Guo T, Wan X, Wang H, Zhu M, Li A, Su N, Shen Y, Mao B, Zhai H (2010). Transcriptome analysis of grain-filling caryopses reveals involvement of multiple regulatory pathways in chalky grain formation in rice. BMC Genom.

[CR43] Livak KJ, Schmittgen TD (2001). Analysis of relative gene expression data using real-time quantitative PCR and the 2(-Delta Delta C(T)) Method. Methods.

[CR44] Lu K, Wu B, Wang J, Zhu W, Nie H, Qian J, Huang W, Fang Z (2018). Blocking amino acid transporter *OsAAP3* improves grain yield by promoting outgrowth buds and increasing tiller number in rice. Plant Biotechnol J.

[CR45] Lu Y, Feng Z, Meng Y, Bian L, Xie H, Mysore KS, Liang J (2020). SLENDER RICE1 and *Oryza sativa* INDETERMINATE DOMAIN2 regulating OsmiR396 are involved in stem elongation. Plant Physiol.

[CR46] Lu YE, Song ZY, Lu K, Lian XM, Cai HM (2012). Molecular characterization, expression and functional analysis of the amino acid transporter gene family (*OsAATs*) in rice. Acta Physiol Plant.

[CR47] Matsushima R, Maekawa M, Kusano M, Kondo H, Fujita N, Kawagoe Y, Sakamoto W (2014). Amyloplast-localized *SUBSTANDARD STARCH GRAIN4* protein influences the size of starch grains in rice endosperm. Plant Physiol.

[CR48] Miyazaki A, Ikeda A, Yonemaru J, Morita S, Yamamoto Y (2018). Relationships among the chalkiness, kernel size and endosperm cell morphology of rice kernels at different spikelet positions within a panicle. Plant Prod Sci.

[CR49] Morita S, Yonemaru J-I, Takanashi J-I (2005). Grain growth and endosperm cell size under high night temperatures in rice (*Oryza sativa* L.). Ann Bot.

[CR50] Nakamura Y, Ono M, Suto M, Kawashima H (2020). Analysis of malto-oligosaccharides and related metabolites in rice endosperm during development. Planta.

[CR51] Nakata M, Fukamatsu Y, Miyashita T, Hakata M, Kimura R, Nakata Y, Kuroda M, Yamaguchi T, Yamakawa H (2017). High temperature-induced expression of rice alpha-amylases in developing endosperm produces chalky grains. Front Plant Sci.

[CR52] Nevame AYM, Emon RM, Malek MA, Hasan MM, Alam MA, Muharam FM, Aslani F, Rafii MY, Ismail MR (2018). Relationship between high temperature and formation of chalkiness and their effects on quality of rice. Biomed Res Int.

[CR53] O’Leary BM (2021) Playing with Pyr: alternate sources of mitochondrial pyruvate fuel plant respiration. 33. 10.1093/plcell/koab147 10.1093/plcell/koab147 PMC8408450 35233626

[CR54] Ohyama A, Tominaga R, Toriba T, Tanaka W (2022). D-type cyclin *OsCYCD3;1* is involved in the maintenance of meristem activity to regulate branch formation in rice. J Plant Physiol.

[CR55] Osugi A, Itoh H, Ikeda-Kawakatsu K, Takano M, Izawa T (2011). Molecular dissection of the roles of phytochrome in photoperiodic flowering in rice. Plant Physiol.

[CR56] Panda BB, Badoghar AK, Sekhar S, Shaw BP, Mohapatra PK (2016). 1-MCP treatment enhanced expression of genes controlling endosperm cell division and starch biosynthesis for improvement of grain filling in a dense-panicle rice cultivar. Plant Sci.

[CR57] Peng B, Kong H, Li Y, Wang L, Zhong M, Sun L, Gao G, Zhang Q, Luo L, Wang G (2014). *OsAAP6* functions as an important regulator of grain protein content and nutritional quality in rice. Nat Commun.

[CR58] Peng C, Wang Y, Liu F, Ren Y, Zhou K, Lv J, Zheng M, Zhao S, Zhang L, Wang C (2014). *FLOURY ENDOSPERM6* encodes a CBM48 domain-containing protein involved in compound granule formation and starch synthesis in rice endosperm. Plant J.

[CR59] Ratseewo J, Warren FJ, Siriamornpun S (2019). The influence of starch structure and anthocyanin content on the digestibility of Thai pigmented rice. Food Chem.

[CR60] Ryoo N, Yu C, Park CS, Baik MY, Park IM, Cho MH, Bhoo SH, An G, Hahn TR, Jeon JS (2007). Knockout of a starch synthase gene *OsSSIIIa/Flo5* causes white-core floury endosperm in rice (*Oryza sativa* L.). Plant Cell Rep.

[CR61] Sandoval G (2012). Lipases and phospholipases: methods and protocols. Methods Mol Biol.

[CR62] She KC, Kusano H, Koizumi K, Yamakawa H, Hakata M, Imamura T, Fukuda M, Naito N, Tsurumaki Y, Yaeshima M (2010). A novel factor *FLOURY ENDOSPERM2* is involved in regulation of rice grain size and starch quality. Plant Cell.

[CR63] Shi X, Tian Q, Deng P, Zhang W, Jing W (2021). The rice aldehyde oxidase *OsAO3* gene regulates plant growth, grain yield, and drought tolerance by participating in ABA biosynthesis. Biochem Biophys Res Commun.

[CR64] Sou SC, Chen WJ, Hsieh W-S, Jeng S-F (2006). Severe obstetric complications and birth characteristics in preterm or term delivery were accurately recalled by mothers. J Clin Epidemiol.

[CR65] Stumpf CR, Moreno MV, Olshen AB, Taylor BS, Ruggero D (2013). The translational landscape of the mammalian cell cycle. Mol Cell.

[CR66] Sun W, Hui Xu X, Lu X, Xie L, Bai B, Zheng C, Sun H, He Y, Xie XZ (2017). The rice phytochrome genes, *PHYA* and *PHYB*, have synergistic effects on anther development and pollen viability. Sci Rep.

[CR67] Suriyasak C, Harano K, Tanamachi K, Matsuo K, Tamada A, Iwaya-Inoue M, Ishibashi Y (2017). Reactive oxygen species induced by heat stress during grain filling of rice (*Oryza sativa* L.) are involved in occurrence of grain chalkiness. J Plant Physiol.

[CR68] Takano M, Inagaki N, Xie X, Kiyota S, Baba-Kasai A, Tanabata T, Shinomura T (2009). Phytochromes are the sole photoreceptors for perceiving red/far-red light in rice. Proc Natl Acad Sci.

[CR69] Takano M, Inagaki N, Xie X, Yuzurihara N, Hihara F, Ishizuka T, Yano M, Nishimura M, Miyao A, Hirochika H, Shinomura T (2005). Distinct and cooperative functions of phytochromes A, B, and C in the control of deetiolation and flowering in rice. Plant Cell.

[CR70] Tang S, Zhang H, Liu W, Dou Z, Zhou Q, Chen W, Wang S, Ding Y (2019). Nitrogen fertilizer at heading stage effectively compensates for the deterioration of rice quality by affecting the starch-related properties under elevated temperatures. Food Chem.

[CR71] Teng X, Zhong M, Zhu X, Wang C, Ren Y, Wang Y, Zhang H, Jiang L, Wang D, Hao Y (2019). *FLOURY ENDOSPERM16* encoding a NAD-dependent cytosolic malate dehydrogenase plays an important role in starch synthesis and seed development in rice. Plant Biotechnol J.

[CR72] Thevenot EA, Roux A, Xu Y, Ezan E, Junot C (2015). Analysis of the human adult urinary metabolome variations with age, body mass index, and gender by implementing a comprehensive workflow for univariate and OPLS statistical analyses. J Proteome Res.

[CR73] Uzair M, Long H, Zafar SA, Patil SB, Chun Y, Li L, Fang J, Zhao J, Peng L, Yuan S (2021). Narrow *Leaf21*, encoding ribosomal protein RPS3A, controls leaf development in rice. Plant Physiol.

[CR74] Wang A, Hou Q, Si L, Huang X, Luo J, Lu D, Zhu J, Shangguan Y, Miao J, Xie Y (2019). The PLATZ transcription factor GL6 affects grain length and number in rice. Plant Physiol.

[CR75] Wang E, Wang J, Zhu X, Hao W, Wang L, Li Q, Zhang L, He W, Lu B, Lin H (2008). Control of rice grain-filling and yield by a gene with a potential signature of domestication. Nat Genet.

[CR76] Wang H, Ham TH, Im DE, Lar SM, Jang SG, Lee J, Mo Y, Jeung JU, Kim ST, Kwon SW (2020). A new SNP in rice gene encoding pyruvate phosphate dikinase (PPDK) associated with floury endosperm. Genes.

[CR77] Wang S, Wu K, Yuan Q, Liu X, Liu Z, Lin X, Zeng R, Zhu H, Dong G, Qian Q (2012). Control of grain size, shape and quality by *OsSPL16* in rice. Nat Genet.

[CR78] Wang X, Zhou W, Lu Z, Ouyang Y, Chol Su O, Yao J (2015). A lipid transfer protein, *OsLTPL36*, is essential for seed development and seed quality in rice. Plant Sci.

[CR79] Wang Y, Xiong G, Hu J, Jiang L, Yu H, Xu J, Fang Y, Zeng L, Xu E, Xu J (2015). Copy number variation at the *GL7* locus contributes to grain size diversity in rice. Nat Genet.

[CR80] Wu M, Ren Y, Cai M, Wang Y, Zhu S, Zhu J, Hao Y, Teng X, Zhu X, Jing R (2019). Rice *FLOURY ENDOSPERM10* encodes a pentatricopeptide repeat protein that is essential for the trans-splicing of mitochondrial nad1 intron 1 and endosperm development. New Phytol.

[CR81] Xi M, Wu W, Xu Y, Zhou Y, Chen G, Ji Y, Sun X (2020). iTRAQ-based quantitative proteomic analysis reveals the metabolic pathways of grain chalkiness in response to nitrogen topdressing in rice. Plant Physiol Biochem.

[CR82] Xiao L, Huang Z, Su Y, Liu Q, Kabir MH (2017). Dynamics of phytohormones and their relationship with chalkiness of early indica rice under different post-anthesis temperature regimes. Bangladesh J Agric Res.

[CR83] Xie Q, Xu J, Huang K, Su Y, Tong J, Huang Z, Huang C, Wei M, Lin W, Xiao L (2021). Dynamic formation and transcriptional regulation mediated by phytohormones during chalkiness formation in rice. BMC Plant Biol.

[CR84] Xing HL, Dong L, Wang ZP, Zhang HY, Han CY, Liu B, Wang XC, Chen QJ (2014). A CRISPR/Cas9 toolkit for multiplex genome editing in plants. BMC Plant Biol.

[CR85] Xu SB, Yu HT, Yan LF, Wang T (2010). Integrated proteomic and cytological study of rice endosperms at the storage phase. J Proteome Res.

[CR86] Yang W, Xu P, Zhang J, Zhang S, Li Z, Yang K, Chang X, Li Y (2022). *OsbZIP60*-mediated unfolded protein response regulates grain chalkiness in rice. J Genet Genom.

[CR87] Yu L, Liu Y, Lu L, Zhang Q, Chen Y, Zhou L, Chen H, Peng C (2017). Ascorbic acid deficiency leads to increased grain chalkiness in transgenic rice for suppressed of L-GalLDH. J Plant Physiol.

[CR88] Yu M, Wu M, Ren Y, Wang Y, Li J, Lei C, Sun Y, Bao X, Wu H, Yang H (2021). Rice *FLOURY ENDOSPERM 18* encodes a pentatricopeptide repeat protein required for 5' processing of mitochondrial nad5 messenger RNA and endosperm development. J Integr Plant Biol.

[CR89] Yu X, Yang T, Qi Q, Du Y, Shi J, Liu X, Liu Y, Zhang H, Zhang Z, Yan N (2021). Comparison of the contents of phenolic compounds including flavonoids and antioxidant activity of rice (*Oryza sativa*) and Chinese wild rice (*Zizania latifolia*). Food Chem.

[CR90] Zhang C, Hao W, Lu Y, Yang Y, Chen Z, Li Q, Fan X, Luo J, Liu Q (2022). A comparative evaluation of the effect of *SSI* and *Wx* allelic variation on rice grain quality and starch physicochemical properties. Food Chem.

[CR91] Zhang H, Tan GL, Wang ZQ, Yang JC, Zhang JH (2009). Ethylene and ACC levels in developing grains are related to the poor appearance and milling quality of rice. Plant Growth Regul.

[CR92] Zhang L, Ren Y, Lu B, Yang C, Feng Z, Liu Z, Chen J, Ma W, Wang Y, Yu X (2016). *FLOURY ENDOSPERM7* encodes a regulator of starch synthesis and amyloplast development essential for peripheral endosperm development in rice. J Exp Bot.

[CR93] Zhang XF, Tong JH, Bai AN, Liu CM, Xiao LT, Xue HW (2020). Phytohormone dynamics in developing endosperm influence rice grain shape and quality. J Integr Plant Biol.

[CR94] Zhao H, Qin Y, Xiao Z, Li Q, Yang N, Pan Z, Gong D, Sun Q, Yang F, Zhang Z (2020). Loss of function of an RNA polymerase III subunit leads to impaired maize kernel development. Plant Physiol.

[CR95] Zhao YF, Peng T, Sun HZ, Teotia S, Wen HL, Du YX, Zhang J, Li JZ, Tang GL, Xue HW, Zhao QZ (2019). miR1432-*OsACOT* (Acyl-CoA thioesterase) module determines grain yield via enhancing grain filling rate in rice. Plant Biotechnol J.

[CR96] Zheng M, Wang Y, Liu X, Sun J, Wang Y, Xu Y, Lv J, Long W, Zhu X, Guo X (2016). The *RICE MINUTE-LIKE1* (*RML1*) gene, encoding a ribosomal large subunit protein L3B, regulates leaf morphology and plant architecture in rice. J Exp Bot.

[CR97] Zhou W, Wang X, Zhou D, Ouyang Y, Yao J (2017). Overexpression of the 16-kDa alpha-amylase/trypsin inhibitor *RAG2* improves grain yield and quality of rice. Plant Biotechnol J.

[CR98] Zhu H, Ai H, Cao L, Sui R, Ye H, Du D, Sun J, Yao J, Chen K, Chen L (2018). Transcriptome analysis providing novel insights for Cd-resistant tall fescue responses to Cd stress. Ecotoxicol Environ Saf.

[CR99] Zhu X, Liang W, Cui X, Chen M, Yin C, Luo Z, Zhu J, Lucas WJ, Wang Z, Zhang D (2015). Brassinosteroids promote development of rice pollen grains and seeds by triggering expression of carbon starved anther, a MYB domain protein. Plant J.

