# Supplementary material for: Transcriptome and Metabolome Analyses Reveals the Pathway and Metabolites of Grain Quality Under Phytochrome B in Rice (Oryza sativa L.)
Source: Rice (N Y). 2022 Oct 27;15:52. doi: 10.1186/s12284-022-00600-5 (PMC9613846; doi:10.1186/s12284-022-00600-5)
Supplement: Supplementary file 1 — Additional file 1. Supplementary Materials and Figures. [file 12284_2022_600_MOESM1_ESM.docx]

**1. Metabolite profiling (MetWare: https://www.metware.cn/)**

The freeze-dried leaf was crushed using a mixer mill (MM 400, Retsch) with a zirconia bead for 1.5 min at 30 Hz. 100 mg powder was weighted and extracted overnight at 4℃ with 0.6 mL 70% aqueous methanol. After centrifugation at 10,000 g for 10 min, the supernatants were filtered (SCAA-104, 0.22 μm pore size; ANPEL, Shanghai, China, http://www.anpel.com.cn/) and analyzed by an LC‐ESI‐MS/MS system The sample extracts were analyzed by an UPLC-ESI-MS/MS system (UPLC, Shim-pack UFLC SHIMADZU CBM30A system, www.shimadzu.com.cn/; MS, Applied Biosystems. 4500 Q TRAP, www.appliedbiosystems.com.cn/). The analytical conditions for the HPLC were: column, ACQUITY UPLC HSS T3 C18 (1.8 µm, 2.1 mm*100 mm) (Waters, https://www.waters.com); solvent system, water (0.04% v/v acetic acid): acetonitrile (0.04% v/v acetic acid); gradient program, 95:5 v/v at 0 min, 5:95 v/v at 11.0 min, 5:95 v/v at 12.0 min, 95:5 v/v at 12.1 min, 95:5 v/v at 15.0 min; flow rate, 0.40 mL min–1; temperature, 40°C; injection volume, 2 μL. The effluent was alternatively connected to an ESI triple‐quadrupole linear ion‐trap (Q TRAP)‐MS. LIT. Triple quadrupole (QQQ) scans were acquired on a triple quadrupole-linear ion trap mass spectrometer (Q TRAP), API 4500 Q TRAP UPLC/MS/MS System, equipped with an ESI Turbo Ion-Spray interface, operating in positive and negative ion mode and controlled by Analyst 1.6.3 software (AB Sciex). The ESI source operation parameters were as follows: ion source, turbo spray; source temperature, 500 °C; ion spray voltage (IS), 5500 V; ion source gas I (GSI), gas II (GSII) and curtain gas (CUR) were set at 50, 60, and 30.0 psi, respectively. The collision gas (CAD) was high. Instrument tuning and mass calibration were performed with 10‐ and 100‐μm polypropylene glycol solutions in QQQ and LIT modes, respectively. The QQQ scans were acquired with collision gas (nitrogen) set to 5 psi according to MRM experiments. DP and CE for individual MRM transitions were performed with further DP and CE optimization. A specific set of MRM transitions was monitored for each period according to the metabolites eluted within the period.

**2. Results**


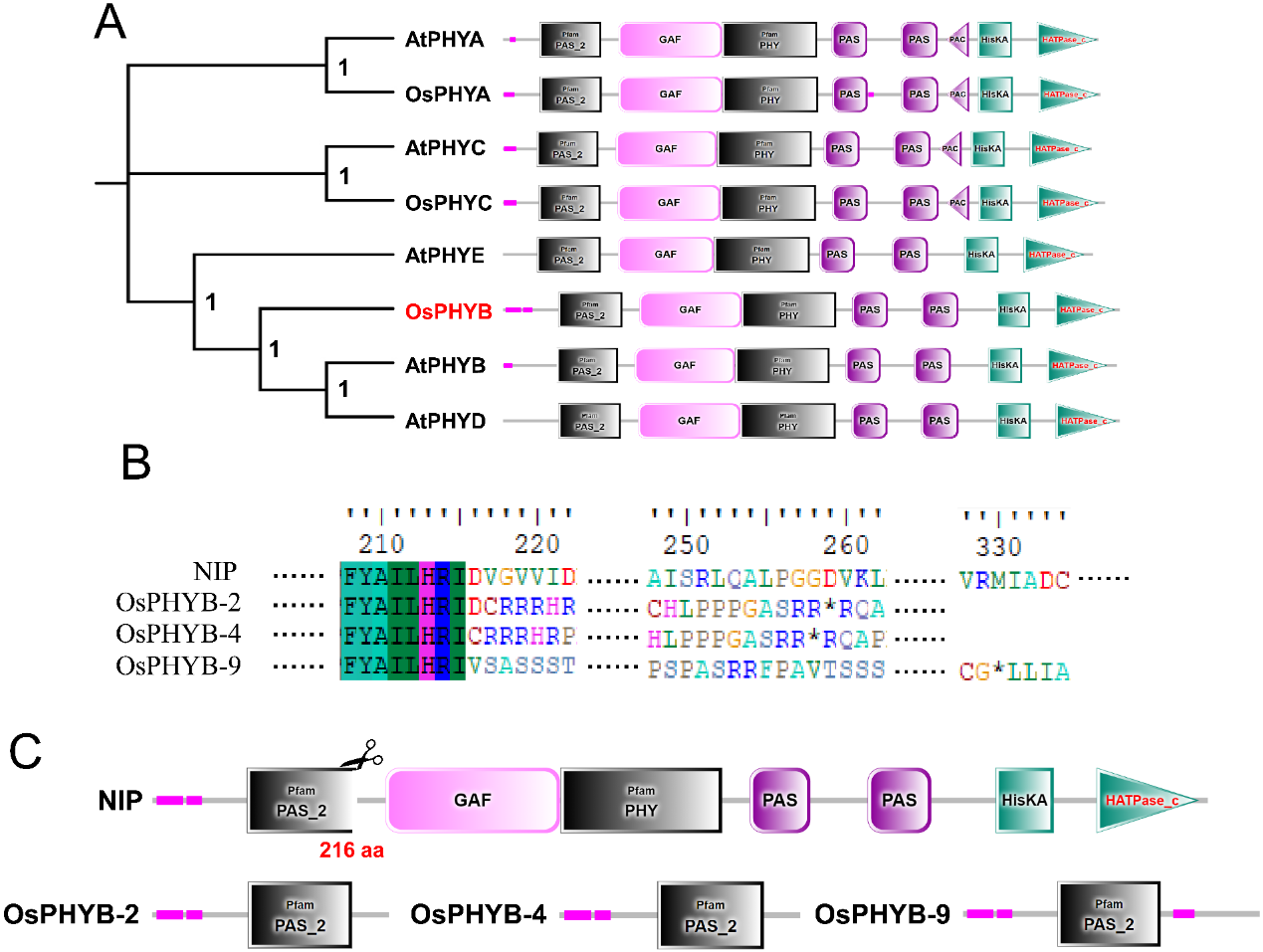


Supplementary Fig. 1. Amino acid structure analysis of *OsPHYB* mutants. (A) Evolutionary analysis of phytochromes in rice and Arabidopsis. (B) Three T3 generation pure and knockout lines of *osphyb* terminated prematurely amino acid translation. (C) SMART (http://smart.embl-heidelberg.de/) protein structure prediction after knockout of the *OsPHYB* target sequence.


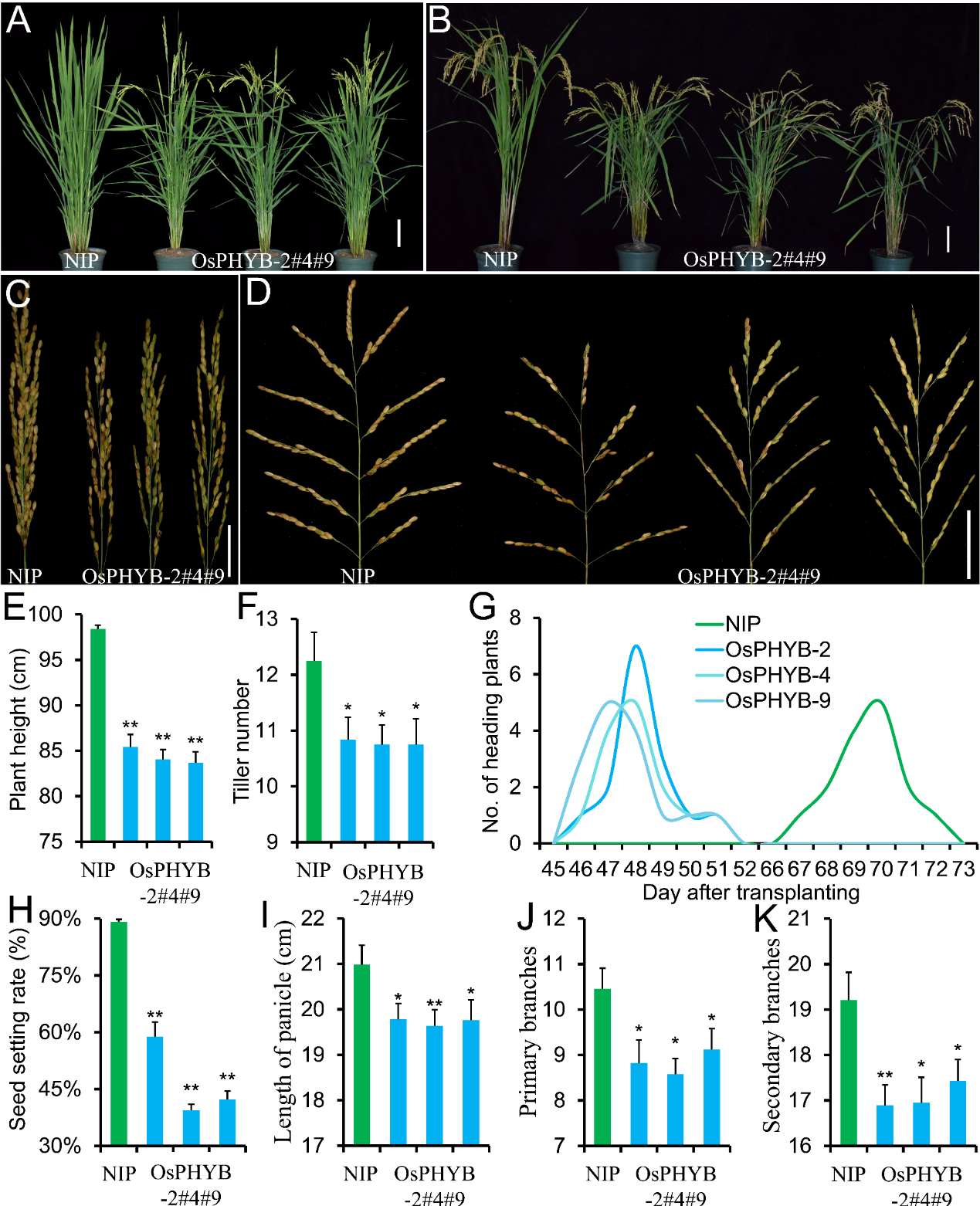


Supplementary Fig. 2. Phenotypes of NIP and *OsPHYB* knockout transgenic plants grown in the field in summer in Zhengzhou. (A, B) Phenotypes of *OsPHYB* knockout lines and NIP whole plant. Bars=10 cm. (C, D) Phenotypes of *OsPHYB* knockout lines and NIP panicle. Bars=5 cm. (E-K) Comparison of three *OsPHYB* knockout lines with the NIP for plant height, tiller number, flowering period, seed setting rate, length of panicle, number of primary and secondary branches. * *P* < 0.05, ** *P* < 0.01.


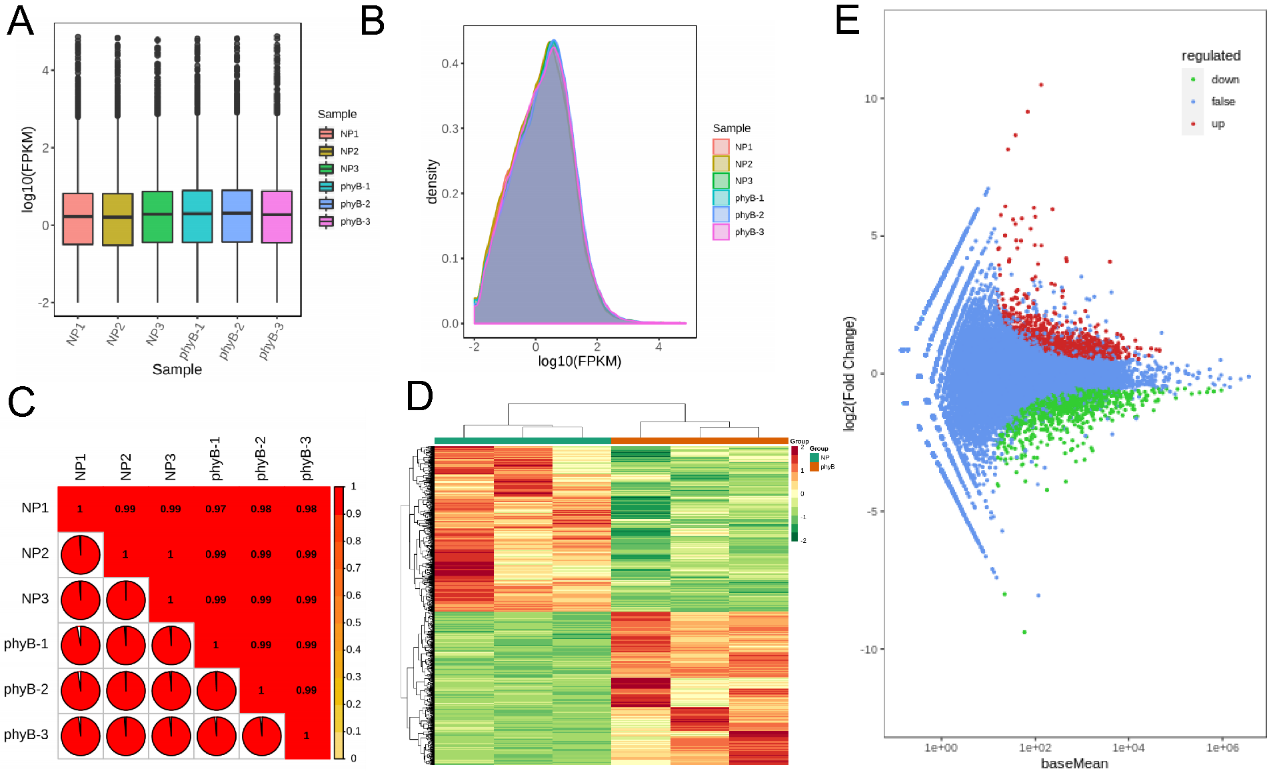


Supplementary Fig. 3. Transcriptome-wide analysis in *osphyb* and NIP. (A) Boxplots of *osphyb* and NIP expression. (B) Distribution of genesexpression density in *osphyb* and NIP. (C) Statistical graph of the correlation between *osphyb* and NIP. (D) Clustering heatmap of differential genes in *osphyb* and NIP. (E) Volcano plot of differential expressed genes between *osphyb* and NIP.


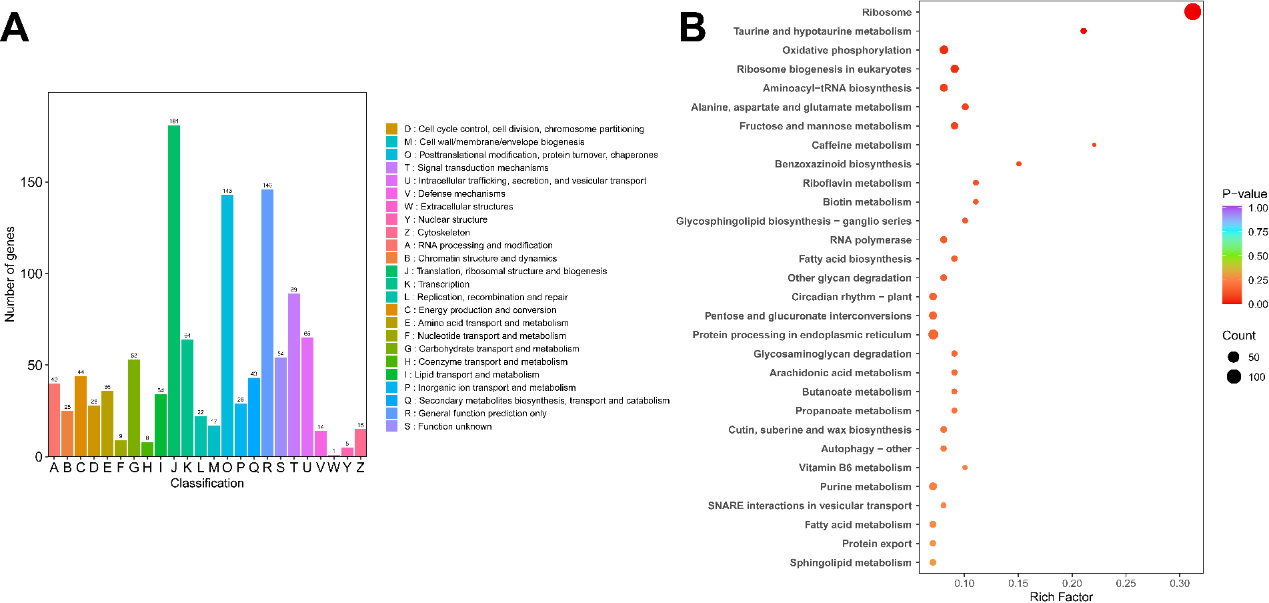


Supplementary Fig. 4. KOG annotation (A) and KEGG pathway enrichment (B) between *osphyb* and NIP.


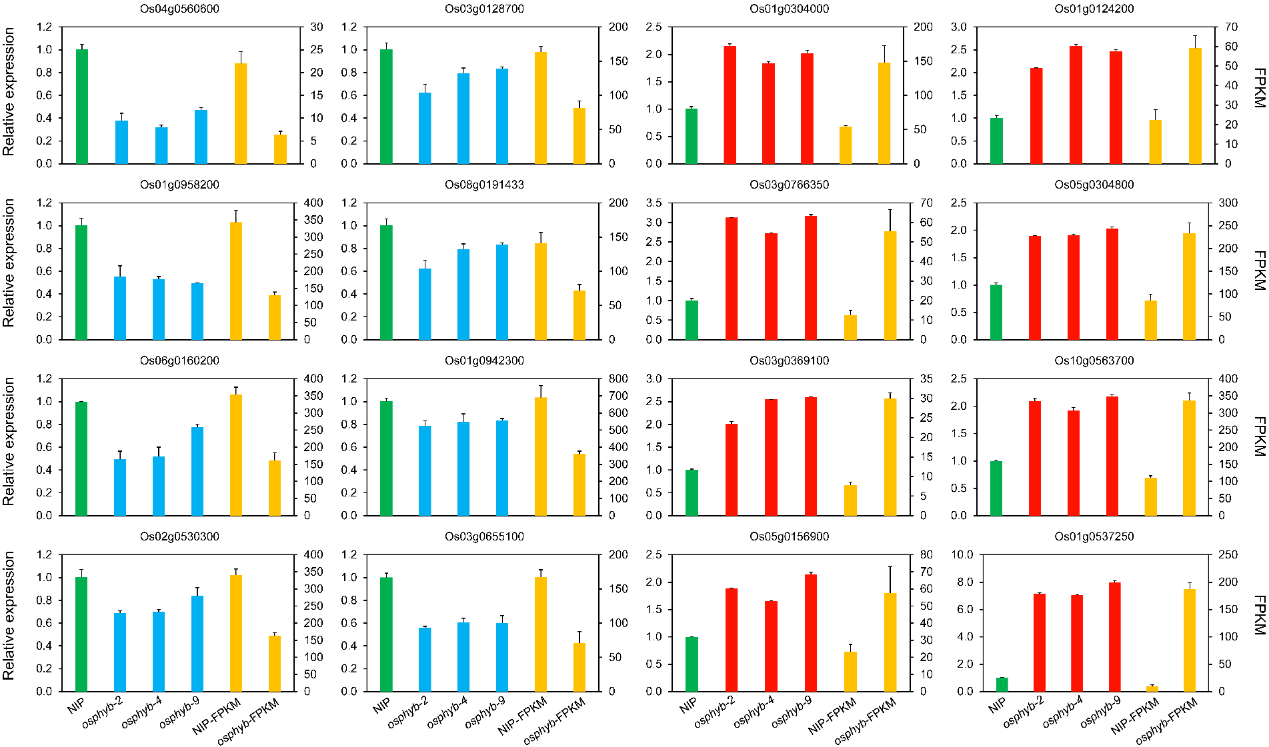


Supplementary Fig. 5. Real-time RT-PCR validation of the RNA-Seq results. Fragments per kilobase of exon per million fragments of mapped reads (FPKM) are shown in yellow.


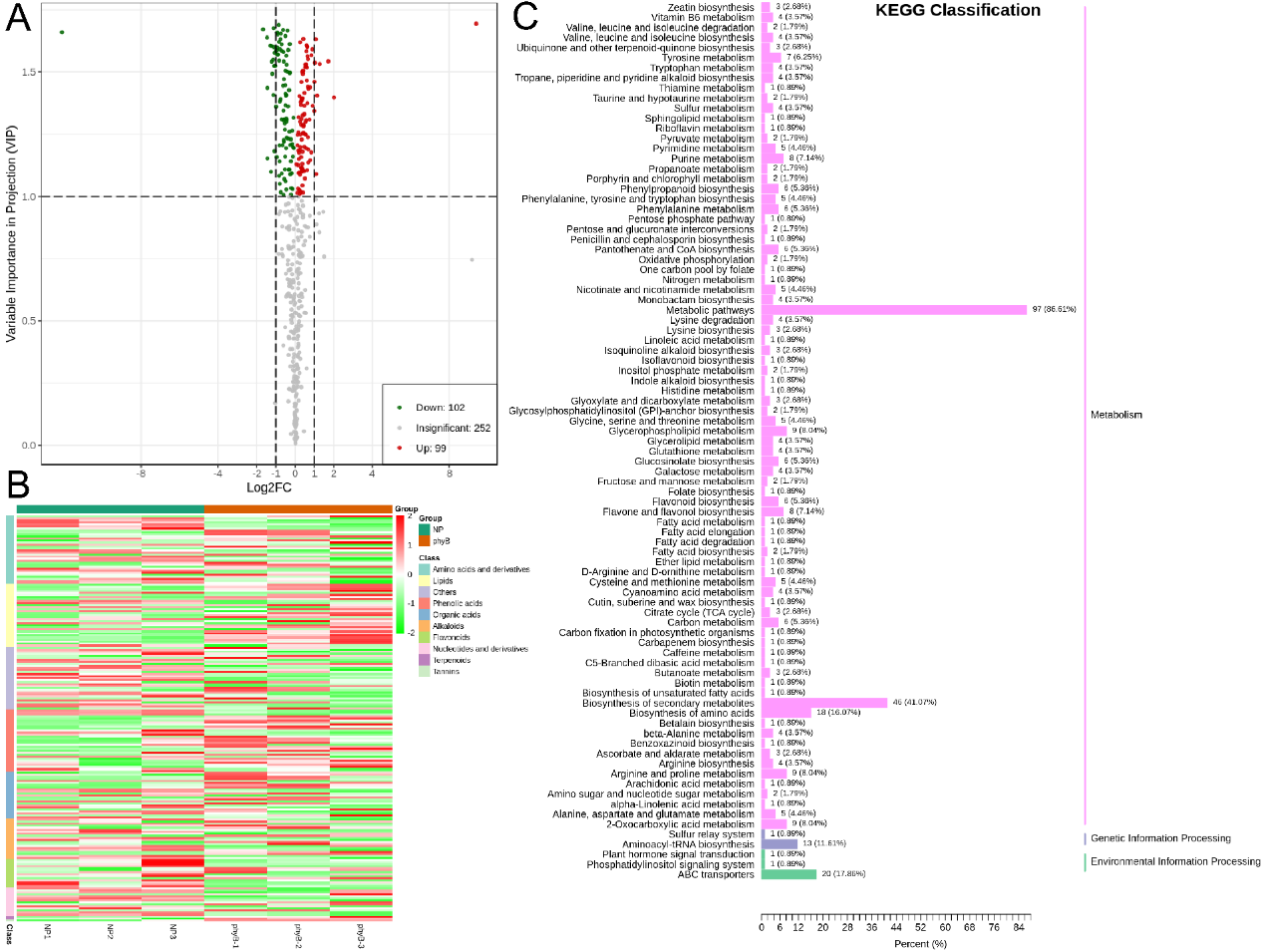


Supplementary Fig. 6. Metabolome-wide analysis in *osphyb* and NIP. (A) Volcano plot of differential metabolites in *osphyb* and NIP. (B) Clustering heatmap of differential metabolites in *osphyb* and NIP. (C) KEGG classification map of differential metabolites in *osphyb* and NIP.


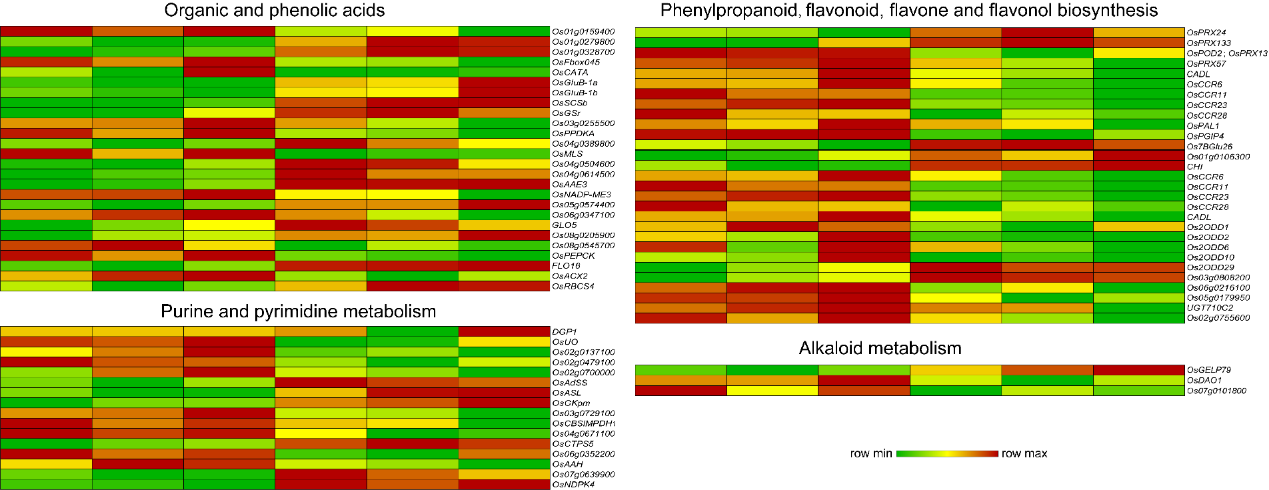


Supplementary Fig. 7. *OsPHYB* regulates the expression levels of various genes involved in organic and phenolic acids, purine and pyrimidine metabolism, phenylpropanoid, flavonoid, flavone and flavonol biosynthesis, and alkaloid metabolism. Each row (6 boxes) represents a DEG. Six boxes in each row represent 3 biological repeats of a gene in NIP (first three boxes) and *osphyb* (last three boxes), respectively. Log_2_ (fold changes) are represented by a colour scale from green (down-regulated expression) to red (up-regulated expression).
